# A Continuous Correlation Between Residual Tumor Volume and Survival Recommends Maximal Safe Resection in Glioblastoma Patients: A Nomogram for Clinical Decision Making and Reference for Non-Randomized Trials

**DOI:** 10.3389/fonc.2021.748691

**Published:** 2021-12-13

**Authors:** Marco Skardelly, Marlene Kaltenstadler, Felix Behling, Irina Mäurer, Jens Schittenhelm, Benjamin Bender, Frank Paulsen, Jürgen Hedderich, Mirjam Renovanz, Jens Gempt, Melanie Barz, Bernhard Meyer, Ghazaleh Tabatabai, Marcos Soares Tatagiba

**Affiliations:** ^1^ Department of Neurosurgery, University Hospital Tuebingen, Eberhard Karls University Tuebingen, Tuebingen, Germany; ^2^ Center for Neuro-Oncology, Comprehensive Cancer Center Tuebingen Stuttgart, University Hospital Tuebingen, Eberhard Karls University of Tuebingen, Tuebingen, Germany; ^3^ Department of Neurology, Eberhard Karls University of Tuebingen, Tuebingen, Germany; ^4^ Department Interdisciplinary Neuro-Oncology, Eberhard Karls University of Tuebingen, Tuebingen, Germany; ^5^ Institute of Pathology and Neuropathology, Division of Neuropathology, University Hospital Tuebingen, Eberhard Karls University Tuebingen, Tuebingen, Germany; ^6^ Department of Neuroradiology, University Hospital Tuebingen, Eberhard Karls University Tuebingen, Tuebingen, Germany; ^7^ University Department of Radiation Oncology, University Hospital Tuebingen, Eberhard Karls University of Tuebingen, Tuebingen, Germany; ^8^ Medistat GmbH, Kiel, Germany; ^9^ Department of Neurosurgery, University Medical Center, Johannes Gutenberg University Mainz, Mainz, Germany; ^10^ Department of Neurosurgery, Klinikum rechts der Isar, Technische Universität München, Munich, Germany

**Keywords:** glioblastoma, extent of resection, residual tumor volume, prognostic survival model, accelerated failure time, nomogram, reference

## Abstract

**Objective:**

The exact role of the extent of resection or residual tumor volume on overall survival in glioblastoma patients is still controversial. Our aim was to create a statistical model showing the association between resection extent/residual tumor volume and overall survival and to provide a nomogram that can assess the survival benefit of individual patients and serve as a reference for non-randomized studies.

**Methods:**

In this retrospective multicenter cohort study, we used the non-parametric Cox regression and the parametric log-logistic accelerated failure time model in patients with glioblastoma. On 303 patients (training set), we developed a model to evaluate the effect of the extent of resection/residual tumor volume on overall survival and created a score to estimate individual overall survival. The stability of the model was validated by 20-fold cross-validation and predictive accuracy by an external cohort of 253 patients (validation set).

**Results:**

We found a continuous relationship between extent of resection or residual tumor volume and overall survival. Our final accelerated failure time model (pseudo R^2^ = 0.423; C-index = 0.749) included residual tumor volume, age, *O^6^-methylguanine-DNA-methyltransferase* methylation, therapy modality, resectability, and ventricular wall infiltration as independent predictors of overall survival. Based on these factors, we developed a nomogram for assessing the survival of individual patients that showed a median absolute predictive error of 2.78 (mean: 1.83) months, an improvement of about 40% compared with the most promising established models.

**Conclusions:**

A continuous relationship between residual tumor volume and overall survival supports the concept of maximum safe resection. Due to the low absolute predictive error and the consideration of uneven distributions of covariates, this model is suitable for clinical decision making and helps to evaluate the results of non-randomized studies.

## Introduction

Glioblastoma (GBM) is a prognostically unfavorable primary brain tumor with an incidence rate of 3.2 per 100,000 population, representing 14.5% of all primary brain tumors (1). The standard of care remains tumor resection followed by radiation therapy with concomitant and adjuvant temozolomide (TMZ) (2).

Several prognostic factors have been described that significantly influence and predict survival, e.g., methylation of the promoter region of the *O^6^-methylguanine-DNA-methyltransferase* (MGMT) gene, extent of resection (EOR), treatment regimen, age, and assessment scores as Karnofsky performance status (KPS) (3–7). However, neurosurgeons and neuro-oncologists can only influence the EOR (8, 9) and the treatment regimen (2) to a limited extent. Although the EOR is one of the key elements of treatment in GBM, its exact role is still controversial due to the lack of prospective randomized clinical trials and contradictory retrospective studies and interpretations (3–5, 10–12). Different thresholds for a clinically significant effect were proposed, ranging from about 70% to complete resection of the contrast-enhancing tumor (3, 5, 10–12). More importantly, based on these results, it was concluded that resection might only be indicated if the respective thresholds can be achieved. In contrast, Marko et al. proposed a continuous relationship of EOR and survival times, showing that any degree of tumor resection is beneficial, and concluded that a maximum safe resection is generally indicated (4). Marko et al. were the first group to present data based on a parametric model of survival analysis, the accelerated failure time (AFT) model, instead of the commonly used semiparametric proportional hazard models. They suggested that their model had better explanatory capacity for survival prediction than other published models based on recursive partitioning analysis or resection thresholds (3, 5, 10–12).

In this study, we wanted to i) validate the concept of a continuous relationship of EOR and survival suggested by the parametric AFT model; ii) extend the introduced AFT model by considering molecular prognostic biomarkers [methylation of MGMT and mutations of *isocitrate dehydrogenase* (IDH)] and radiological/surgical predictors for survival prediction; iii) compare the explanatory power of the AFT model with different Cox proportional hazard models; iv) provide a reliable nomogram for predicting survival; and v) evaluate the model for clinical applicability in an independent cohort.

## Methods

### Study Design

This is a retrospective multicenter cohort study addressing the relationship of EOR and overall survival (OS) in adult patients with newly diagnosed IDH wild-type GBM. The models were developed on the basis of a patient cohort of one of the three involved centers, which served as a training set (*n* = 303). The other patients were combined as a cohort to externally validate the final statistical models (validation set, *n* = 253). The clinical endpoint OS was evaluated by univariate and multivariable Cox regression analyses and AFT model. The different models were cross-validated and compared by their coefficients of determination (pseudo R^2^) and concordance indices (C-indices). Based on the β-coefficients from the AFT model, a score was derived from convincing predictors by means of a nomogram, and a score-related prediction model for OS was developed.

### Data Collection and Study Population

We included all adult patients (age ≥ 18 years) with newly diagnosed GBM treated at one of the study centers from January 2006 to December 2014. The institutional ethics committees of three universities approved the study. The following variables were obtained for each patient: gender, age at diagnosis, molecular markers (mutations of IDH and methylation of MGMT), KPS, tumor location, preoperative tumor volume, residual tumor volume (RTV), white matter infiltration related to ventricles (contrast-enhanced tumor infiltration of ventricle wall: yes or no), eloquent brain regions (dominant side of Wernicke’s and Broca’s speech area and inferior parietal lobule “Geschwind’s” region; both sides of the primary motor, sensory, and visual cortices), postoperative deficits on the day of discharge (median d6), treatment modality, use of steroids, time of surgery, time of tumor progression according to Response Assessment in Neuro-Oncology (RANO), death, and last visit. MGMT was determined locally in the different centers without central assessment. MRI within 72 h after surgery assessed RTV by comparing T1-weighted images with and without contrast enhancement. We used Brainlab (BrainLAB AG, Feldkirchen, Germany) for volumetric analyses. Patients with an IDH mutation, incomplete data sets (e.g., missing postoperative MRI and missing molecular markers), or participation in therapy arms of clinical trials were excluded.

### Statistical Analyses

Only patients with complete data sets were included in the analyses; patients with incomplete data sets were excluded. First, we performed univariate Cox regressions to identify potential variables that have an impact on OS. Variables were analyzed using the full spectrum of continuous variables but were also categorized (age, KPS, EOR, and RTV) by classification and regression tree (CART) analyses or by common thresholds according to literature: age (≤50 vs. >50 to ≤70 vs. >70 years); KPS (≥90 vs. <90); EOR (100%, 98%, 95%, and 80%), and RTV (0, ≤1, 1–10, and >10 cm^3^). We introduced a new variable called “resectability”. We stratified patients into “good” or “bad” resectable with respect to tumor locations that were significantly associated with worse survival in univariate Cox regressions. Tumors were defined as bad resectable if the tumor was in a diencephalic location, a thalamic location, the basal ganglia, or brain stem or if the tumor was multicenter; otherwise, it is was defined as good resectable. Multicollinearity between the identified risk factors was excluded.

Variables that showed hazard ratios (HRs) with *p*-values ≤0.1 were used to perform multistep Cox regressions with bidirectional elimination. The proportional hazard assumption was confirmed by analyzing Schoenfeld residuals and Rho statistics. Models were internally validated by 20-fold cross-validation. The goodness of fit was assessed by estimating the Cox–Snell pseudo R^2^, which corresponds to the level of variation that is explained by the regression model. Furthermore, the C-index was determined, which is a generalization of the area under the receiver operating characteristic curve that measures the model’s discrimination power (see document, **Supplementary File 1**, which explains the whole development of the statistical models, Model design “1.1–1.3,” pp. 1–7).

The most promising EOR model was determined by several multivariable Cox regressions considering different absolute and relative RTV thresholds (see document, **Supplementary File 1**, Appendix—Comparison of different EOR models “4.1–4.9,” pp. 22–29).

Log-logistic AFT models were performed based on selected factors from Cox models. The assumption of a log-logistic distribution was tested and confirmed. The AFT model was also internally validated by 20-fold cross-validation. Residuals were calculated for the comparison of the predicted and observed OS (see document, **Supplementary File 1**, Model design 1.4, pp. 8–11). The final AFT model with categorical variables was used to create a score from a nomogram based on the β-coefficients, which was again validated by log-logistic regression (see document, **Supplementary File 1**, Scoring for survival “2,” pp. 12–14). Finally, AFT models of a) categorical predictors and b) the derived score were validated by an external patient cohort by comparing the mean and median absolute predictive error (APE), the Cox–Snell pseudo R^2^, and C-index of models and external validations (see document, **Supplementary File 1**, Model validation on external data “3,” pp. 15–18). JMP 12.2 (SAS Institute Inc., Cary, NC; https://www.jmp.com/en_us/home.html) and some functions from R (13) and R package rms (14) were used for the statistical analyses.

## Results

### Patients and Overall Survival

**Figure 1:**
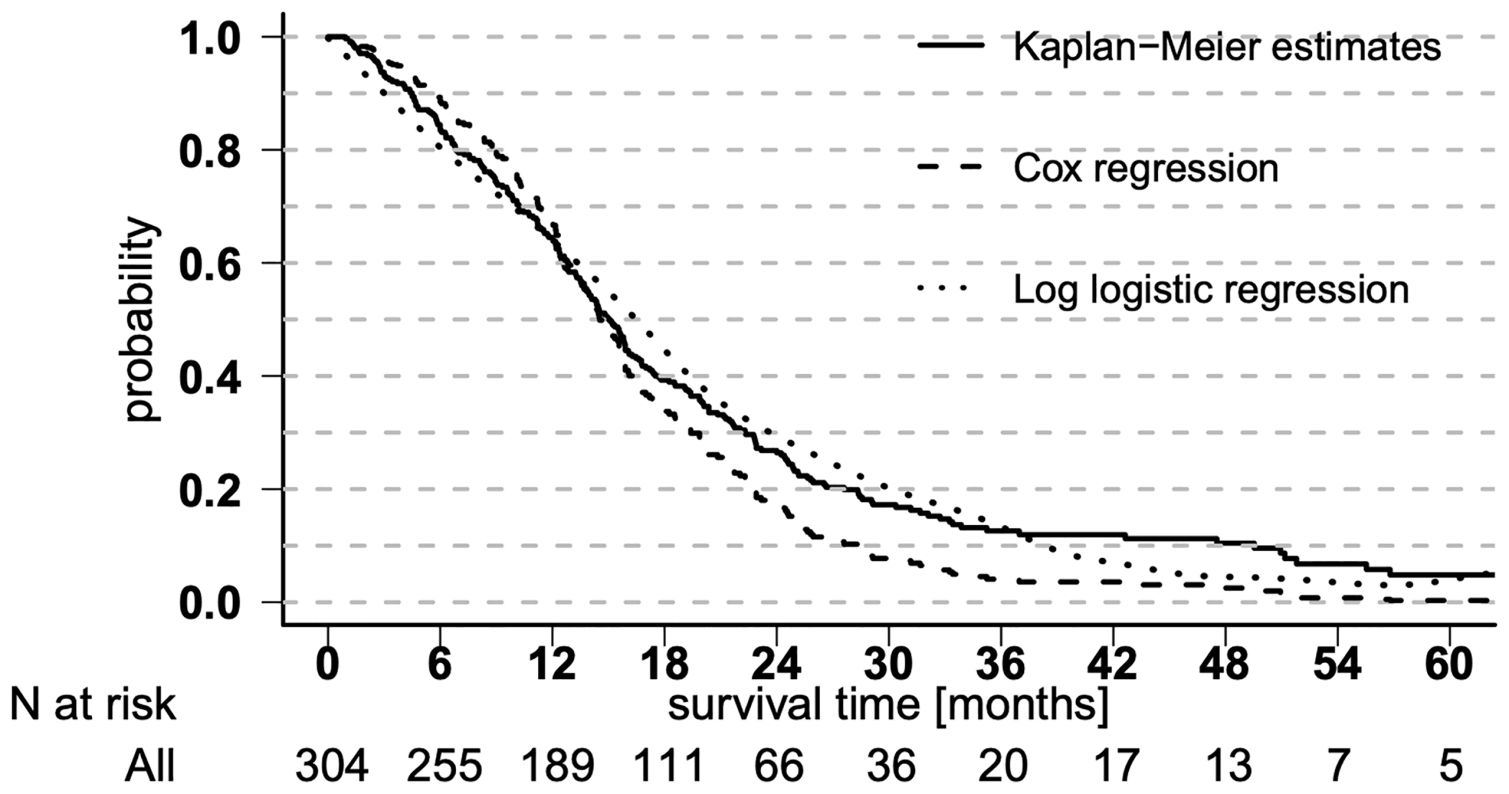
Overall survival curves. Overall survival shown in Kaplan–Meier estimates and derived from Cox regression and log-logistic regression.

Out of 392 IDH wild-type GBM patients who were treated in our hospitals between 2006 and 2014, 303 patients had complete data sets and were available as a training set for multivariable regressions. Eighty-nine patients were excluded because of missing MRI data (*n* = 48), inclusion in study arms of prospective studies (*n* = 36), and missing MGMT status (*n* = 13). At the time of analysis, 254 patients had died (84%), 26 were still alive (8.5%), and 23 were lost to follow-up (7.5%). Patient characteristics are presented in **Supplementary File 2**. The median OS was 15.0 months (95% CI 13–16), and the median time to progression was 8.4 months (95% CI 7.4–9.2). Estimations of OS rates are shown in **Figure 1** as Kaplan–Meier, Cox regression, and log-logistic regression survival curves; and the table in **Supplementary File 3** illustrates the OS Kaplan–Meier estimates. There is a trend in regression curves towards underestimating longer survival compared with Kaplan–Meier, especially in Cox regression.

### Relationship Between Residual Tumor Volume and Overall Survival

**Figure 2:**
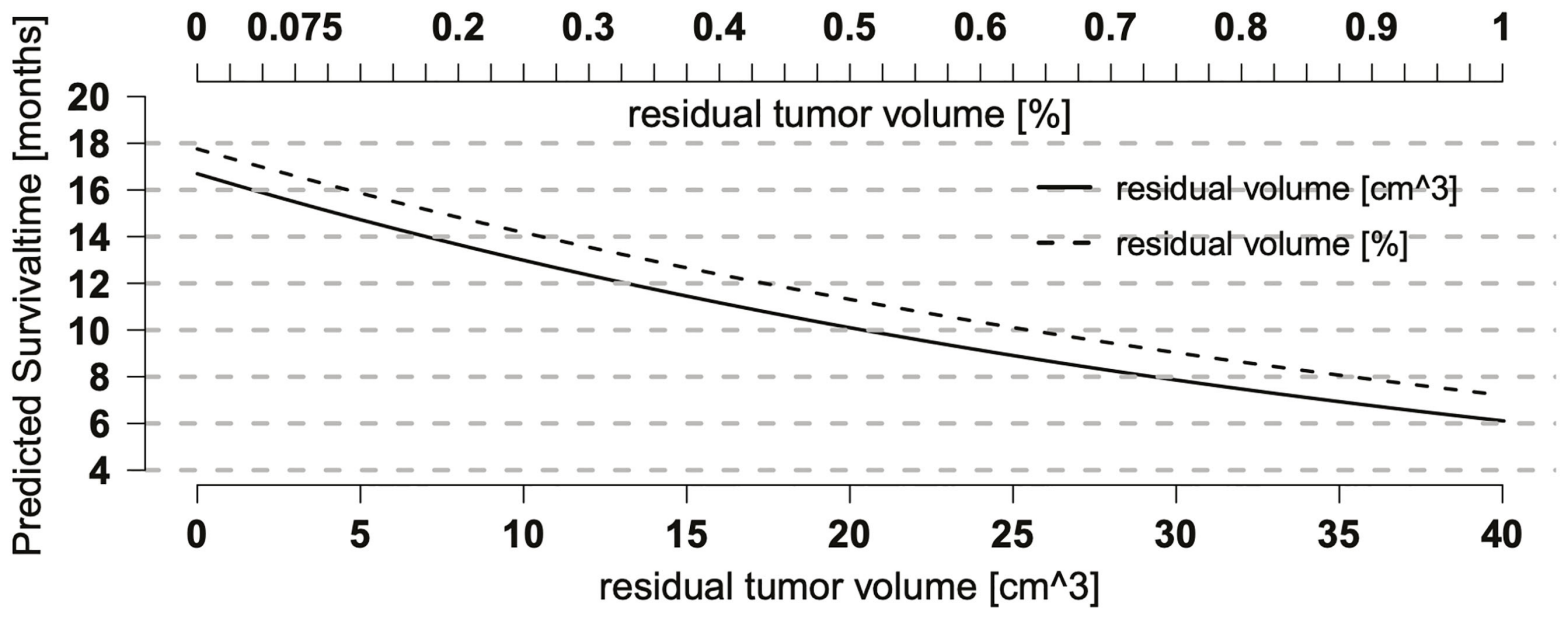
Relationship between residual tumor volume (RTV) and overall survival (OS). Relationship between predicted OS and RTV or extent of resection (EOR) as single predictors in a log-logistic regression model. Both curves show a continuous, nearly linear relationship and run in parallel with a better prognosis for relative RTV (EOR), suggesting that preoperative tumor size also may have an effect on OS.

The parametric AFT model allows the prediction and visualization of the relationship of clinically relevant parameters in addition to point estimates for individual survival times. **Figure 2** illustrates the continuous almost linear relationship between EOR and the median predicted OS. **Table 1** shows the parameters of the logistic regression model. The coefficient of RTV (−0.0127) can be used to calculate the estimated OS as a function of residual tumor size. For example, an RTV of 10 cm^3^ leads to a shortening in survival time by a factor of 0.88 [exp (−0.0127 × 10)].

**Table 1:** Log-logistic regression model.

Characteristic	Final AFT model		
	Coefficient^§^	SE	*p*
Intercept	4.1685	0.3978	<0.0001
RTV (cm^3^)	−0.0127	0.0032	<0.0001
Age (years)	−0.0183	0.0050	0.0002
MGMT, unmethylated	−0.4316	0.0849	<0.0001
Radiotherapy	0.1295	0.1616	0.4229
Radiochemotherapy	0.3884	0.1761	0.0274
Resectability, bad	−0.3321	0.1095	0.0024
Infiltration of vent. Wall	−0.3478	0.0823	<0.0001
Log(scale)	−0.9653	0.0528	<0.0001
Pseudo R^2^	0.404		
C-index	0.748		

RTV, residual tumor volume; MGMT, O^6^-methylguanine-DNA-methyltransferase; AFT, accelerated failure time.

^§^ The coefficients in the log-logistic model detect acceleration or deceleration in survival times [acceleration factor (AF)]. The transformation with the exponential function leads to values <1 (delay—disadvantageous) or >1 (acceleration—advantageous). For example, in a patient with a tumor of 80 cm^3^ with exp(−0.0127 × 80) = 0.36, expected survival is shortened to 36% with biopsy and 94% with subtotal resection, with an RTV of 5 cm^3^ with exp(−0.0127 × 5) = 0.94, compared with complete resection.

### Model Development and Validation

Univariate Cox regressions suggested age, RTV, EOR, methylation of MGMT, KPS, therapy modality, resectability, and white matter infiltration relating to ventricles to be significant predictors of OS. Eloquence, the use of preoperative steroids, and recurrent surgery were, i.a., not significant factors for OS. Multivariable Cox and log-logistic regressions confirmed continuous variables age and RTV and methylation of MGMT, postoperative therapy modality, resectability, and white matter infiltration relating to ventricles as possible predictors of OS. In contrast, KPS was excluded because it had no independent effect on OS. Age and RTV were grouped into three categories. For the complete model development, see document, **Supplementary File 1**, which explains the whole development of the statistical models.

The final AFT model was tested against the null model (χ^2^ = 166.09; <0.0001, **Table 2**).

**Table 2:** Final survival model of the prognostic score.

Characteristic	Final AFT model			
	Coefficient^§^	SE	AF	*p*
Intercept	3.2403	0.2046		<0.0001
RTV >10 to ≤20 cm^3^	−0.4717	0.1905	0.624	0.0133
RTV >20 cm^3^	−0.7840	0.1498	0.457	<0.0001
Age >50 to ≤70	−0.3057	0.1169	0.734	0.0089
Age >70	−0.4798	0.1624	0.619	0.0031
MGMT, unmethylated	−0.4131	0.0834	0.662	<0.0001
Radiotherapy	0.1512	0.1570	1.170	0.3356
Radiochemotherapy	0.4905	0.1697	1.633	0.0039
Resectability, bad	−0.2272	0.1066	0.797	0.0330
Infiltration of vent. wall	−0.3274	0.0810	0.721	<0.0001
Log(scale)	−0.9845	0.0527		<0.0001
Pseudo R^2^	0.423			
C-index	0.749			

RTV, residual tumor volume; MGMT, O^6^-methylguanine-DNA-methyltransferase; AFT, accelerated failure time.

^§^ The coefficients in the log-logistic model detect acceleration or deceleration in survival times [acceleration factor (AF)]. The transformation with the exponential function leads to values <1 (delay—disadvantageous) or >1 (acceleration—advantageous). For example, the factor MGMT with exp(−0.4131) = 0.66 is associated with a survival time for unmethylated versus methylated shortened by a factor of 0.66.

The model demonstrated a pseudo R^2^ of 0.423, which is the amount of variation of OS that is explained by our regression model, thereby explaining its goodness of fit. The C-index, which is the proportion of all pairs of cases where the case with empirically shorter survival times also has a higher predicted risk (hazard) and thus can be interpreted as a measure of the predictive power of the model, was 0.749, indicating a good model. The internal validation by 20-fold cross-validation shows after correction for optimism a pseudo R^2^ of 0.428 and a C-index of 0.755, which is very close to the final model demonstrating the stability of the estimates. The median deviation of 0.95 months (mean 0.30 months) is low; i.e., the model applies to the observed data. However, individual deviations can be quite high, and there is a trend towards underestimating longer survival. For external validity assessment, a novel external data set of 253 patients was available, of which 191(76%) had died at the time of analysis and 62 (24%) were still alive or lost to follow-up. Snell’s pseudo R^2^ of this model was 0.271 and C-index 0.686, resulting in a median APE of 2.63 months (mean: 1.81 months).

The parametric AFT model allows the prediction and visualization of the relationship of clinically relevant parameters in addition to point estimates for individual survival times. **Figure 2** illustrates the continuous almost linear relationship between EOR and the median predicted OS.

### The Nomogram Established

**Figure 3:**
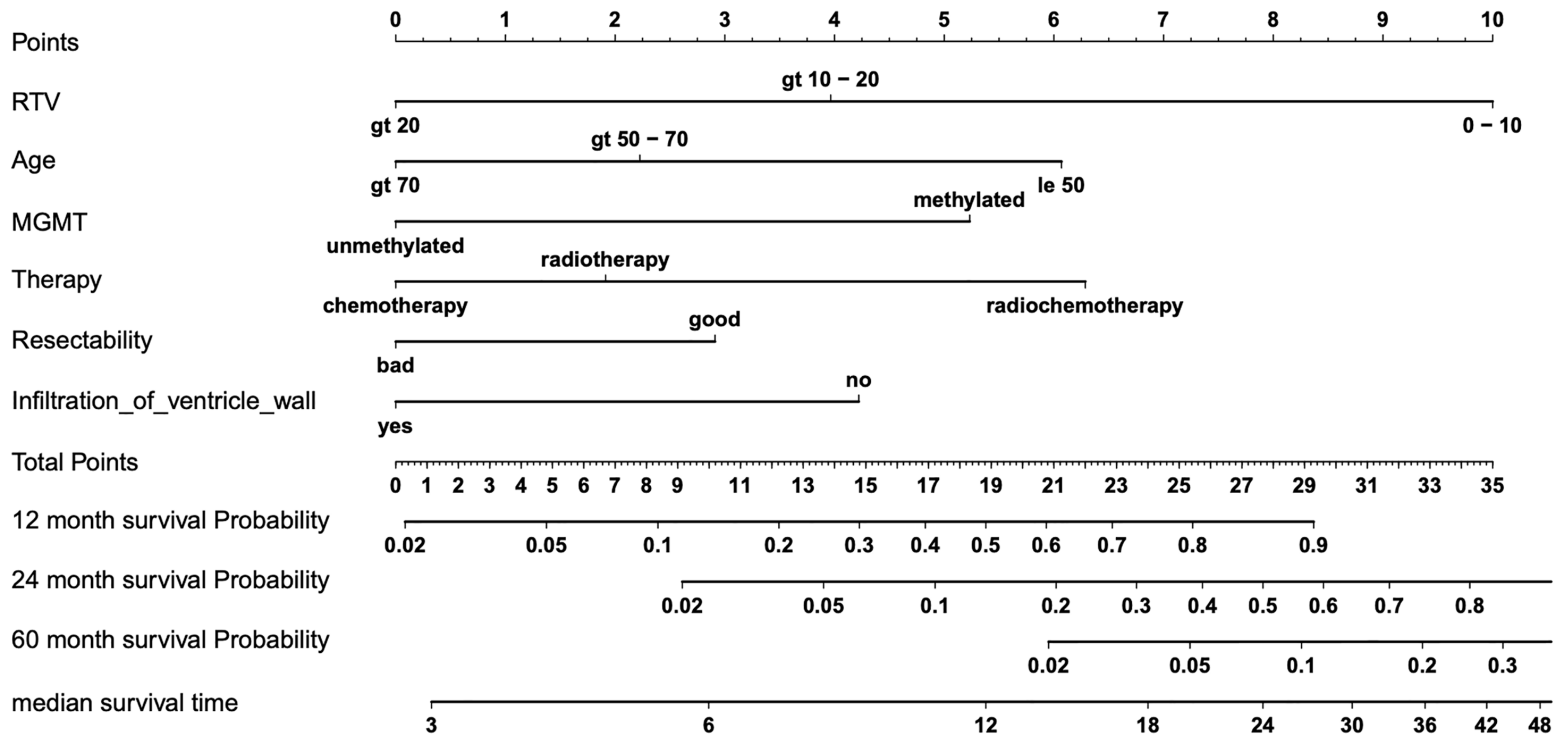
Nomogram for predicting overall survival (OS). Final nomogram predicting individual median survival times and 12, 24, and 60 months of survival probability based on six predictors of OS, which add up in a summary score from 0 (worst) to 34 (best) total points; for examples, see table, **Supplementary File 4**, with four clinical cases of nomogram-predicted survival versus actual survival.

**Figure 4:**
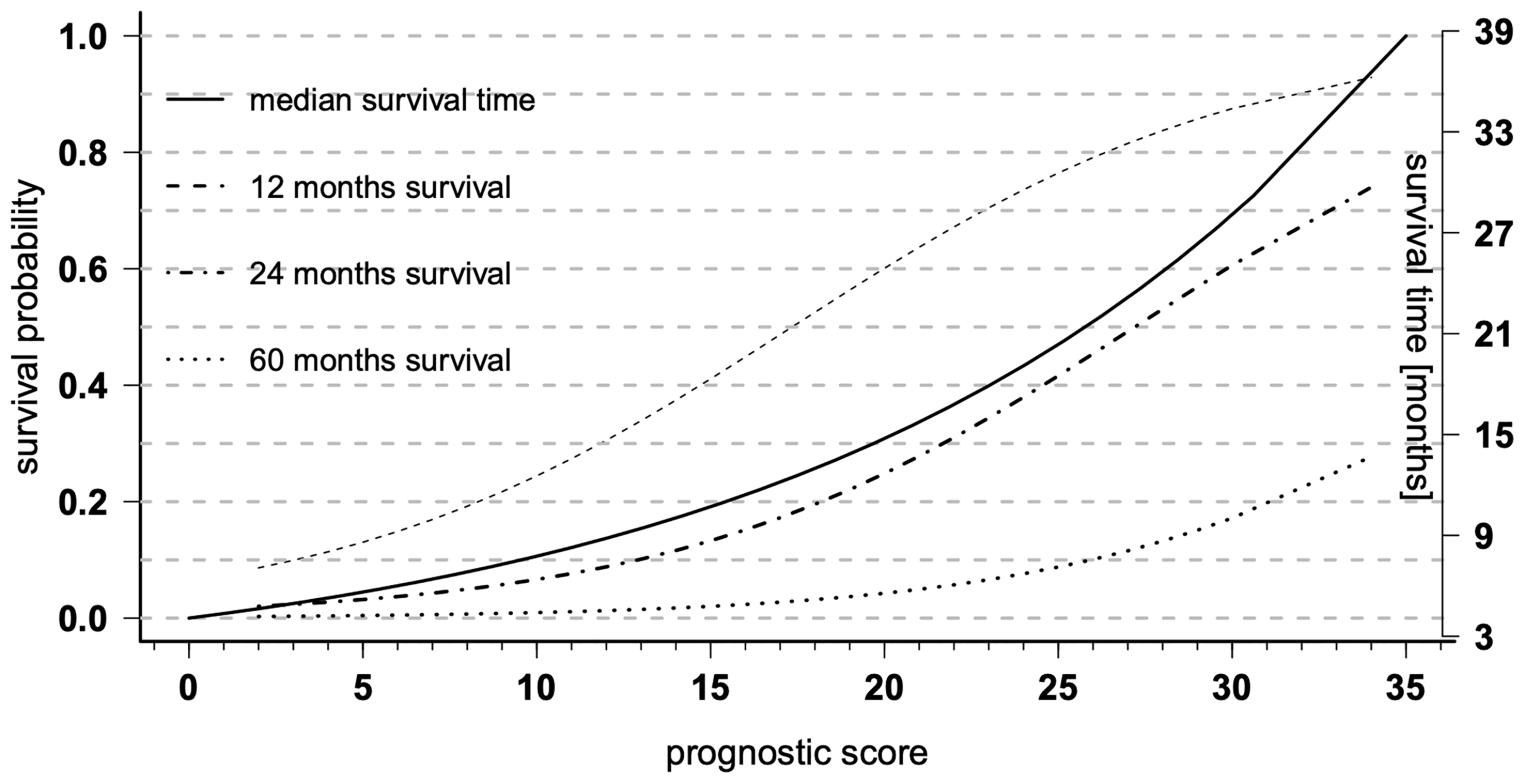
Prognostic diagrams for overall survival (OS). Prognostic diagrams for the median OS (full line, right y-axis) and 12, 24, and 60 months (dashed lines, left y-axis) survival probability based on the total prognostic scores of the nomogram.

A nomogram to estimate individual survival probabilities was built using the final AFT model (**Figure 3**). Median survival and survival rates at 12, 24, and 60 months are obtained from drawing a perpendicular line from the “Total points” axis to the outcome axes. Up to 34 points are possibly given with the best score of 34 and the worst score of 0 points. Alternatively, the score can also be calculated by summing up the score value for each variable (see **Table 3**, showing the scores of each category of predictors for OS) and reading out the survival probabilities in **Figure 4**. For clinical examples, see **Supplementary File 4**.

**Table 3:** Scoring for survival.

Risk factors for overall survival			
**Residual tumor volume (cm^3^)**	≤10	>10 to ≤ 20 cm^3^	>20
	**10**	**4**	**0**
**Age (years)**	≤50	>50 to ≤ 70	>70
	**6**	**2**	**0**
**MGMT**	Methylated		Unmethylated
	**5**		**0**
**Therapy modality**	Radiochemotherapy	Radiotherapy	Chemotherapy
	**6**	**2**	**0**
**Resectability**	Good		Bad
	**3**		**0**
**Infiltration of ventricular wall**	No		Yes
	**4**		**0**

The “worst” score (with the worst forecast) is thus 0; the best value is 34. The AFT model (χ^2^ = 166.95; p < 0.0001, see document, **Supplementary File 1**, Scoring for survival “2,” p. 15) of the score demonstrated a pseudo R^2^ of 0.423 and a C-index of 0.748 and was validated on the independent data set with a pseudo R^2^ of 0.239 and a C-index of 0.678 resulting in a median APE of 2.78 months (mean: 1.83 months).

MGMT, O^6^-methylguanine-DNA-methyltransferase.

## Discussion

We evaluated the effects of EOR on survival using non-parametric and parametric survival models, demonstrated the advantages and limitations of the AFT model, and provided an improved nomogram-based prediction model. We also found a continuous relationship between EOR and survival, as suggested by Marko et al. (4). By additionally considering molecular markers (IDH and MGMT), resectability, and the extent of white matter infiltration, we were able to improve the AFT model (pseudo R^2^ = 0.31 to pseudo R^2^ = 0.42) and to reduce the APE by about 1.8 months from a median of 4.42 months to a median of 2.63 months compared with the model of Marko et al. (4). We developed a clinically applicable nomogram to predict survival times (C-index = 0.69) with an APE of a median of 2.78 months or a mean of 1.8 months. The developed models show an improvement of about 40% compared with the most promising currently established model (4). Finally, we present that despite the further improvement of the model to estimate individual survival times, the model is still not sufficient to reliably predict individual survival times but is suitable to facilitate clinical decision making and to predict the mean/median survival in smaller groups of patients, e.g., for phase 1/2 trials.

### Predictors of Overall Survival in Glioblastoma

To estimate the actual impact of the different predictors of survival, all covariates that affect survival must be identified and integrated into the multivariable regression. Among numerous clinical, radiological, and molecular factors (**Supplementary File 2**), only seven factors demonstrated a significant effect on OS in univariate regressions and were reduced to six factors in our final multivariable models (**Tables 1**, **2**). Our data confirm that younger age at diagnosis, higher EOR or lower RTV, methylated MGMT, and postoperative combined radiochemotherapy or radiotherapy compared with chemotherapy are favorable predictors of survival as previously suggested (4, 7, 15). In contrast to Gittleman et al., KPS and gender had no independent impact on OS in our patient cohort in accordance with the observations of Marko et al. and Gorlia et al. (4, 7, 15). In univariate regression, KPS was also a significant predictor of OS. The multivariate regression showed that KPS was not an independent predictor of survival when the other variables in our model were included. Because it is a multidimensional process, we cannot explain the reason for this precisely but can only speculate. Because most of the other identified variables (age, therapy, extent of resection, MGMT status) are generally also taken into account in other studies, we might speculate that the variable “resectability” newly introduced in our model is responsible. If the differences observed in KPS are explained to a large extent by "resectability", i.e., tumor location, the independent effect of KPS on OS might no longer be large enough to exert a statically significant independent influence on OS. In addition, tumor infiltration beyond the white matter into the ventricular wall had an unfavorable independent effect on OS. This was also observed by Wangaryattawanich et al., who also found deep white matter invasion and ependymal extension as significant predictors of OS (16). In addition, we have introduced a new factor called “resectability” of contrast-enhancing tumor. Tumors stratified as poorly resectable have been shown to be an independent unfavorable predictor of OS in our cohort. Tumor expansion in classic eloquent regions was not a significant predictor of OS, as observed by others (3, 17) because safe tumor removal can now be ensured in these regions through the introduction of electrophysiology and awake surgery.

### Tumor Volume and Survival

We confirmed the continuous inverse relationship between RTV and OS (4), which means that any degree of resection has a benefit of survival. This is in contrast to most studies published in the last two decades that identified different thresholds for a beneficial role of EOR directing different clinical recommendations (3, 5, 10–12). The observed differences in these studies are likely due to the different underlying statistical models. Non-parametric models (e.g., Cox regression) that are commonly used forfeit information by defining dichotomous or categorical thresholds and calculating the median survival by considering the population medians of covariates with semi-quantitative hazards. Interestingly, Lacroix et al. and Grabowski et al. already showed continuous relationships between median OS and increasing thresholds (85%–100%) of EOR (10) or decreasing thresholds of RTVs (25–1 cm^3^) (17). Instead, Marko et al. used parametric log-logistic regression modeling, which uses the full information of metric data, enabling individual point estimations of survival and providing visualization of the probabilistic relationship of RTV and survival, a concept that was also applied in this study.

The concept of a continuous relationship between RTV and survival rather than postulated thresholds is also supported by the observation that postoperative RTV, determined as gadolinium enhancement within 24–72 h after surgery in all studies, reflects not the true tumor volume. Increasing evidence suggests that tumor volume in GBM is not restricted to gadolinium enhancement (18–20). Suchorska et al. showed that the biological tumor volume (BTV) determined by *O*-(2-[^18^F]fluoroethyl)-l-tyrosine PET (^18^FET-PET) can be much larger than the volume of gadolinium enhancement and is associated with survival times. They showed that despite complete resection of contrast enhancement, up to 9.5-cm^3^ BTV could still be detected (19). This has also been supported by studies using 5-aminolevulinic acid (5-ALA) for glioma surgery demonstrating tumor infiltration beyond the gadolinium enhancement in MRI (18). Roessler et al. postulated that 5-ALA is more sensitive for RTV than ^18^FET-PET, meaning that GBM extends even beyond BTV in ^18^FET-PET (20). These data suggest that the postulated thresholds based on resection of partial tumor volume are unlikely clinically relevant. Surgeries in patients who were classified as complete resection (5), gross total resection (GTR) of >98% (10), GTR of >78% (3), GTR of >70% (12, 18), etc., have likely more RTV than expected, but patients did, however, benefit from tumor resections. Clinically, these considerations speak against refusing surgery due to the impossibility of obtaining a specific EOR and support the concept of maximum safe resection. This means that surgery is also indicated even in cases of expansive diseases, where only partial tumor resection is safely achievable.

We improved the predictive accuracy of our final regression model and our simplified score model by about 40% compared with the currently established model (4). Although the mean/median APE is small (2.78/1.8 months), individual predictions are still not recommended, as individual deviations can be very high (see document, **Supplementary File 1**, which explains the whole development of the statistical models, Model design, 1.4, p. 11). In contrast, the low APE in our model could be helpful in estimating the effect of therapies in unrandomized studies by considering the combined effect of covariates for each patient and thus compensating for the uneven distribution of risk factors in the different trial groups. This is of particular importance since unrandomized and unstratified retrospective or small prospective phase 1/2 studies do not serve to demonstrate the efficacy of new therapies; patients’ covariate risk factors are often unbalanced, distorting the interpretation of survival times.

### Limitations and Strengths of the Study

The main limitation of this study is the retrospective nature; e.g., clinical data as KPS or neurological deficits were collected through medical records and not according to a defined protocol, and MGMT was determined locally without central assessment. The recently identified biomarker CDKN2A, which has been shown to be associated with OS in GBM (21), was not available for analysis. Patients were neither randomized nor stratified by the other predictors of OS to assess the effects of EOR or RTV on OS. However, a prospective study dealing with this question, i.e., randomizing the EOR, would not be ethically acceptable. We consider the unequal distributions of the other covariates through multivariable analysis. After the development of our model, we have internally demonstrated the stability of our model (C-index 0.75) by cross-validation and validated the predictive power and adaptability by an external independent patient cohort. For model and nomogram development, our patient cohorts covered the entire spectrum of clinical GBM cases without limitations of general performance status (i.e., KPS), age, RTV, or postoperative therapy compared with the developed nomograms from specific patient cohorts of prospective clinical trials (7, 15). Another limitation might be the heterogeneity of the patients and data as assessed by the different study centers. At the same time, however, this represents a strength of the study, as it shows the generalizability of the model. However, all patients included in the model come from three German specialized academic centers, which may limit the transfer of the model to other patient cohorts, e.g., from non-academic centers or from other countries.

## Conclusions

We found a continuous relationship between RTV and OS that supports the concept of maximum safe resection. By considering molecular and radiological markers, we improved the predictive accuracy of previous models by about 40% compared with the most promising established model and developed a clinical applicable score. The developed nomogram helps to estimate the expected survival and the benefit of a more radical surgery. This can be of help to the treating physicians in advising the patients and relatives in the decision for surgery. Nevertheless, individual predictions should only be made with caution on the basis of this model due to the possible high individual deviations. Yet our statistical model could be a very useful tool to estimate the survival effect of retrospective or small prospective phase I/II studies since the median/mean APE is low.

## Data Availability Statement

The raw data supporting the conclusions of this article will be made available by the authors, without undue reservation.

## Ethics Statement

The studies involving human participants were reviewed and approved by Ethik-Kommission an der Medizinischen Fakultät der Eberhard-Karls-Universität und am Universitätsklinikum Tübingen. Written informed consent for participation was not required for this study in accordance with the national legislation and the institutional requirements.

## Author Contributions

Conception and experimental design: MS and MT. Data acquisition: MK, FB, IG-T, JS, BB, FP, MR, JG, and MB. Analysis: MS and JH. Interpretation of the data: MS, MK, JG, MR, JH, MT, and BM. Drafting: MS, GT, and JH. Revision: MK, FB, IG-T, JS, BB, FP, JH, MR, JG, MB, BM, GT, and MT. All authors contributed to the article and approved the submitted version.

## Funding

We acknowledge support by the Open Access Publishing Fund of the University of Tübingen.

## Conflict of Interest

The authors declare that the research was conducted in the absence of any commercial or financial relationships that could be construed as a potential conflict of interest.

## Publisher’s Note

All claims expressed in this article are solely those of the authors and do not necessarily represent those of their affiliated organizations, or those of the publisher, the editors and the reviewers. Any product that may be evaluated in this article, or claim that may be made by its manufacturer, is not guaranteed or endorsed by the publisher.

## Abbreviations

AFT, accelerated failure time; APE, absolute predictive error; CART, classification and regression tree; EOR, extent of resection; GBM, glioblastoma; IDH, *isocitrate dehydrogenase*; KPS, Karnofsky performance status; MGMT, *O^6^-methylguanine-DNA-methyltransferase*; OS, overall survival; RTV, residual tumor volume; TMZ, temozolomide.

## References

[1] Ostrom QT Patil N Cioffi G Waite K Kruchko C Barnholtz-Sloan JS. CBTRUS Statistical Report: Primary Brain and Other Central Nervous System Tumors Diagnosed in the United States in 2013-2017. Neuro-Oncology (2020) 22:iv1–96. doi: 10.1093/neuonc/noaa200 33123732 PMC7596247

[2] Stupp R Mason WP van den Bent MJ Weller M Fisher B Taphoorn MJB. Radiotherapy Plus Concomitant and Adjuvant Temozolomide for Glioblastoma. N Engl J Med (2005) 352:987–96. doi: 10.1056/nejmoa043330 15758009

[3] Sanai N Polley M-Y McDermott MW Parsa AT Berger MS. An Extent of Resection Threshold for Newly Diagnosed Glioblastomas. J Neurosurg (2011) 115:3–8. doi: 10.3171/2011.2.jns10998 21417701

[4] Marko NF Weil RJ Schroeder JL Lang FF Suki D Sawaya RE. Extent of Resection of Glioblastoma Revisited: Personalized Survival Modeling Facilitates More Accurate Survival Prediction and Supports a Maximum-Safe-Resection Approach to Surgery. J Clin Oncol: Off J Am Soc Clin Oncol (2014) 32:774–82. doi: 10.1200/jco.2013.51.8886 PMC4876349 24516010

[5] Kreth FW Thon N Simon M Westphal M Schackert G Nikkhah G. Gross Total But Not Incomplete Resection of Glioblastoma Prolongs Survival in the Era of Radiochemotherapy. Ann Oncol (2013) 24:3117–23. doi: 10.1093/annonc/mdt388 24130262

[6] Hegi ME Diserens A-C Gorlia T Hamou M-F de Tribolet N Weller M. MGMT Gene Silencing and Benefit From Temozolomide in Glioblastoma. N Engl J Med (2005) 352:997–1003. doi: 10.1056/NEJMoa043331 15758010

[7] Gorlia T van den Bent MJ Hegi ME Mirimanoff RO Weller M Cairncross JG. Nomograms for Predicting Survival of Patients With Newly Diagnosed Glioblastoma: Prognostic Factor Analysis of EORTC and NCIC Trial 26981-22981/CE.3. Lancet Oncol (2008) 9:29–38. doi: 10.1016/S1470-2045(07)70384-4 18082451

[8] Senft C Bink A Franz K Vatter H Gasser T Seifert V. Intraoperative MRI Guidance and Extent of Resection in Glioma Surgery: A Randomised, Controlled Trial. Lancet Oncol (2011) 12:997–1003. doi: 10.1016/s1470-2045(11)70196-6 21868284

[9] Stummer W Pichlmeier U Meinel T Wiestler OD Zanella F Reulen H-J. Fluorescence-Guided Surgery With 5-Aminolevulinic Acid for Resection of Malignant Glioma: A Randomised Controlled Multicentre Phase III Trial. Lancet Oncol (2006) 7:392–401. doi: 10.1016/s1470-2045(06)70665-9 16648043

[10] Lacroix M Abi-Said D Fourney DR Gokaslan ZL Shi W DeMonte F. A Multivariate Analysis of 416 Patients With Glioblastoma Multiforme: Prognosis, Extent of Resection, and Survival. J Neurosurg (2001) 95:190–8. doi: 10.3171/jns.2001.95.2.0190 11780887

[11] Li YM Suki D Hess K Sawaya R. The Influence of Maximum Safe Resection of Glioblastoma on Survival in 1229 Patients: Can We do Better Than Gross-Total Resection? J Neurosurg (2016) 124:977–88. doi: 10.3171/2015.5.jns142087 26495941

[12] Chaichana KL Jusue-Torres I Navarro-Ramirez R Raza SM Pascual-Gallego M Ibrahim A. Establishing Percent Resection and Residual Volume Thresholds Affecting Survival and Recurrence for Patients With Newly Diagnosed Intracranial Glioblastoma. Neuro-Oncology (2013) 16:113–22. doi: 10.1093/neuonc/not137 PMC3870832 24285550

[13] team RC. R: A Language and Environment for Statistical Computing. Available at: https://www.R-project.org/.

[14] Harrell FE Jr. Rms: Regression Modeling Strategies. . R Package Version 5.1-1. Available at: https://CRAN.R-project.org/package=rms.

[15] Gittleman H Lim D Kattan MW Chakravarti A Gilbert MR Lassman AB. An Independently Validated Nomogram for Individualized Estimation of Survival Among Patients With Newly Diagnosed Glioblastoma: NRG Oncology RTOG 0525 and 0825. Neuro-Oncology (2016) 19(5):669–77. doi: 10.1093/neuonc/now208 PMC5464437 28453749

[16] Wangaryattawanich P Hatami M Wang J Thomas G Flanders A Kirby J. Multicenter Imaging Outcomes Study of The Cancer Genome Atlas Glioblastoma Patient Cohort: Imaging Predictors of Overall and Progression-Free Survival. Neuro-Oncology (2015) 17:1525–37. doi: 10.1093/neuonc/nov117 PMC4648306 26203066

[17] Grabowski MM Recinos PF Nowacki AS Schroeder JL Angelov L Barnett GH. Residual Tumor Volume Versus Extent of Resection: Predictors of Survival After Surgery for Glioblastoma. J Neurosurg (2014) 121:1115–23. doi: 10.3171/2014.7.jns132449 25192475

[18] Molina ES Schipmann S Stummer W. Maximizing Safe Resections: The Roles of 5-Aminolevulinic Acid and Intraoperative MR Imaging in Glioma Surgery—Review of the Literature. Neurosurg Rev (2019) 42 (2):197–208. doi: 10.1007/s10143-017-0907-z 28921173 PMC6502775

[19] Suchorska B Jansen NL Linn J Kretzschmar H Janssen H Eigenbrod S. Biological Tumor Volume in 18FET-PET Before Radiochemotherapy Correlates With Survival in GBM. Neurology (2015) 84:710–9. doi: 10.1212/WNL.0000000000001262 25609769

[20] Roessler K Becherer A Donat M Cejna M Zachenhofer I. Intraoperative Tissue Fluorescence Using 5-Aminolevolinic Acid (5-ALA) Is More Sensitive Than Contrast MRI or Amino Acid Positron Emission Tomography ((18)F-FET PET) in Glioblastoma Surgery. Neurological Res (2012) 34:314–7. doi: 10.1179/1743132811Y.0000000078 22449387

[21] Lu VM O’Connor KP Shah AH Eichberg DG Luther EM Komotar RJ. The Prognostic Significance of CDKN2A Homozygous Deletion in IDH-Mutant Lower-Grade Glioma and Glioblastoma: A Systematic Review of the Contemporary Literature. J Neuro Oncol (2020) 148:221–9. doi: 10.1007/s11060-020-03528-2 32385699

