# Supplementary material for: A Continuous Correlation Between Residual Tumor Volume and Survival Recommends Maximal Safe Resection in Glioblastoma Patients: A Nomogram for Clinical Decision Making and Reference for Non-Randomized Trials
Source: Front Oncol. 2021 Dec 13;11:748691. doi: 10.3389/fonc.2021.748691 (PMC8711700; doi:10.3389/fonc.2021.748691)
Supplement: Supplementary file 1 [file Presentation_1.pdf]

Supplementary File 1 **Statistics supplements**

1. MODEL DESIGN 1
1.1 COX-REGRESSION – FULL MODEL (KPS INCLUDED) 2
1.2 COX-REGRESSION – REDUCED MODEL (KPS EXCLUDED) 3
1.3 COX-REGRESSION – REDUCED CATEGORICAL MODEL 5
1.4 LOG-LOGISTIC REGRESSION - REDUCED MODEL 7
1.5 LOG-LOGISTIC REGRESSION - REDUCED CATEGORICAL MODEL 8

2. SCORING FOR SURVIVAL 10

PREDICTION FROM THE COEFFICIENTS OF THE MODEL 12

PREDICTION OF THE LOG-LOGISTIC SCORE-MODEL 12

3. MODEL VALIDATION ON EXTERNAL DATA 13
3.1 VALIDATION OF EXTERNAL PATIENT COHORT 13

4. APPENDIX - COMPARISON OF DIFFERENT EOR MODELS 16
4.1 CURRENT MODEL (CATEGORICAL MODEL – 3 RESECTION CATEGORIES, ABSOLUTE) 16
4.2 CATEGORICAL MODEL – 2 RESECTION CATEGORIES, ABSOLUTE 18
4.3 CATEGORICAL MODEL – 2 RESECTION CATEGORIES, ABSOLUTE 18
4.4 CATEGORICAL MODEL – 3 RESECTION CATEGORIES, ABSOLUTE 19
4.5 CATEGORICAL MODEL – 3 RESECTION CATEGORIES, ABSOLUTE 20
4.6 CATEGORICAL MODEL – 5 RESECTION CATEGORIES, ABSOLUTE 21
4.7 CATEGORICAL MODEL – 2 RESECTION CATEGORIES, RELATIVE IN PERCENT 22
4.8 CATEGORICAL MODEL – 3 RESECTION CATEGORIES, RELATIVE IN PERCENT 23
4.9 CATEGORICAL MODEL – 2 RESECTION CATEGORIES, RELATIVE IN PERCENT 24

1. Model design

Three hundred and three cases, 254 uncensored and 49 censored, were available for the modeling including 7 potential predictors of overall survival: extent of resection, age, MGMT-status, therapy modality, Karnofsky performance scale, white matter infiltration related to ventricular wall and resectability.

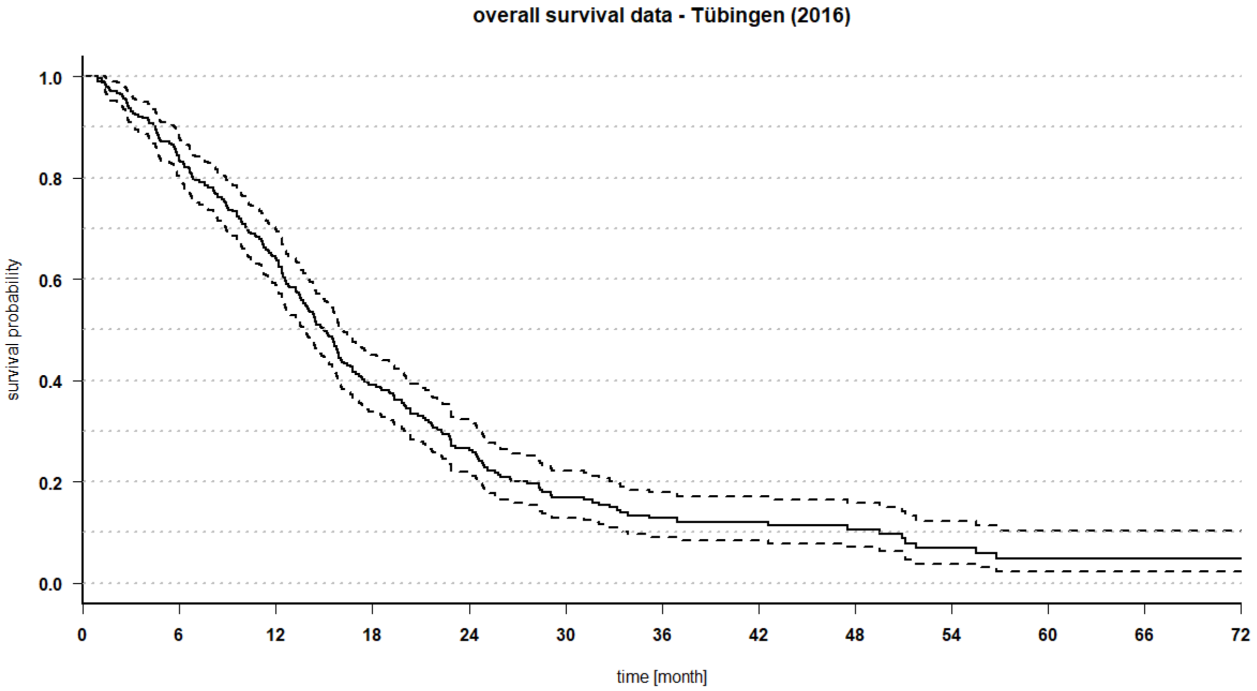

The null model describes the variability (uncertainty) of overall survival without considering influencing variables. It represents the reference for the assessment of the following regression models.

1.1 Cox-Regression – full model (KPS included)

The full Cox regression model is calculated including all 7 predictors identified by univariable analyses:

	coef	exp(coef)	se(coef)	z	Pr(>|z|)
**rtv**	0.023462	1.023739	0.004525	5.185	2.16e-07 ***
**age**	0.029617	1.030060	0.007951	3.725	0.000195 ***
**mgmt, unmethylated**	0.733100	2.081524	0.152230	4.816	1.47e-06 ***
**radiotherapy**	-0.372242	0.689187	0.271748	-1.370	0.170748
**radiochemotherapy**	-0.824541	0.438436	0.292178	-2.822	0.004772 **
**resectability, bad**	0.406133	1.501003	0.179290	2.265	0.023499 *
**kps<90**	0.076495	1.079497	0.138335	0.553	0.580284
**infiltration of vent. wall**	0.460048	1.584151	0.141147	3.259	0.001117 **

Estimated hazard ratios (95%-CI):

	exp(coef)	exp(-coef)	lower .95	upper .95
**rtv**	1.0237	0.9768	1.0147	1.0329
**age**	1.0301	0.9708	1.0141	1.0462
**mgmt, unmethylated**	2.0815	0.4804	1.5446	2.8052
**radiotherapy**	0.6892	1.4510	0.4046	1.1740
**radiochemotherapy**	0.4384	2.2808	0.2473	0.7773
**resectability, bad**	1.5010	0.6662	1.0563	2.1330
**kps90ge 90**	1.0795	0.9264	0.8231	1.4157
**infiltration of vent. wall**	1.5842	0.6313	1.2013	2.0890

Concordance= 0.746 (se = 0.021)
R^2= 0.393

Pseudo R^2 indicates the contribution to likelihood from the null model, which can be explained by the model. The **concordance index (C-index)** is the proportion of all pairs of cases where the case with empirically shorter survival times also has a higher predicted risk (hazard) and thus can be interpreted as a measure of the predictive power of the model. The C index (comparable to the AUC in the ROC analysis) should be greater than 70% for a good model (conversion of Somer's Dxy in the (cross) validation: C = Dxy / 2 + 0.5).

The Cox regression model assumes proportional risks (hazards) over time. This assumption can be checked by the **Rho statistic** or graphically by the **Schoenfeldt residuals**. The residuals to the individual model components should be constantly near the zero line.

Test of proportional hazard assumption in Cox regression model:

	rho	chisq	p
**rtv**	0.099014	2.21e+00	0.1370
**age**	-0.061983	9.42e-01	0.3319
**mgmt, unmethylated**	0.000575	9.57e-05	0.9922
**radiotherapy**	-0.062833	1.15e+00	0.2840
**radiochemotherapy**	-0.041225	4.69e-01	0.4935
**resectability, bad**	-0.075095	1.53e+00	0.2154
**kps<90**	-0.023548	1.57e-01	0.6921
**infiltration of vent. wall**	-0.154153	6.22e+00	0.0126

Schoenfeldt residual plots:

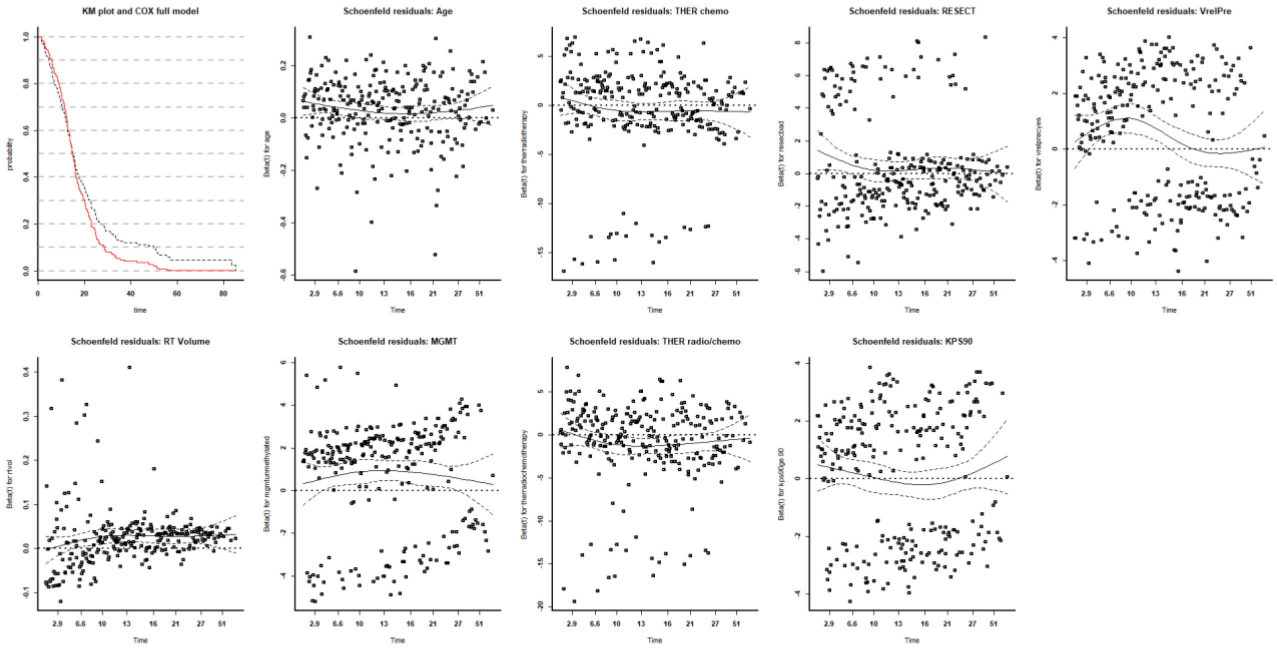

The assumption of proportional hazards is violated only in the *infiltration of ventricle wall* factor. Overall, the survival probability in the Cox model is underestimated relating to Kaplan-Meier estimates.

<u>k-fold cross-validation of Cox regression model (internal):</u>

**index**	**orig**	**training**	**test**	**optimism**	**index corrected**	**n**
Dxy	0.4917	0.4930	0.4354	0.0575	**0.4341**	20
R^2	0.3928	0.3939	0.3734	0.0205	**0.3724**	20

The cross-validation checks the stability of the estimate internally (based on modeling data).

1.2 Cox-Regression – reduced model (KPS excluded)

The reduced model is calculated including 6 predictors identified by univariable analyses without KPS:

	**coef**	**exp(coef)**	**se(coef)**	**z**	**Pr(>|z|)**
**rtv**	0.023679	1.023961	0.004499	5.263	1.42e-07 ***
**age**	0.029860	1.030311	0.007951	3.756	0.000173 ***
**mgmt, unmethylated**	0.722134	2.058822	0.150793	4.789	1.68e-06 ***
**radiotherapy**	-0.374676	0.687512	0.271476	-1.380	0.167543
**radiochemotherapy**	-0.837836	0.432646	0.291262	-2.877	0.004020 **
**resectability, bad**	0.417342	1.517922	0.177989	2.345	0.019039 *
**infiltration of vent. wall**	0.469327	1.598917	0.140089	3.350	0.000808 ***

Estimated hazard ratios (95%-CI):

	**exp(coef)**	**exp(-coef)**	**lower .95**	**upper .95**
**rtv**	1.0240	0.9766	1.0150	1.0330
**age**	1.0303	0.9706	1.0144	1.0465
**mgmt, unmethylated**	2.0588	0.4857	1.5320	2.7668
**radiotherapy**	0.6875	1.4545	0.4038	1.1705
**radiochemotherapy**	0.4326	2.3114	0.2445	0.7657
**resectability, bad**	1.5179	0.6588	1.0709	2.1516

**infiltration of vent. wall**	1.5989	0.6254	1.2150	2.1041

Concordance= 0.746 (se = 0.021)
R^2= 0.392

<u>Test of proportional hazard assumption in Cox regression model:</u>

	**rho**	**chisq**	**p**
**rtv**	0.09696	2.11840	0.1455
**age**	-0.06357	0.98972	0.3198
**mgmt, unmethylated**	0.00268	0.00201	0.9642
**radiotherapy**	-0.06290	1.14960	0.2836
**radiochemotherapy**	-0.04025	0.44864	0.5030
**resectability, bad**	-0.07812	1.63761	0.2007
**infiltration of vent. wall**	-0.15939	6.53434	0.0106

Schoenfeldt residual plots:

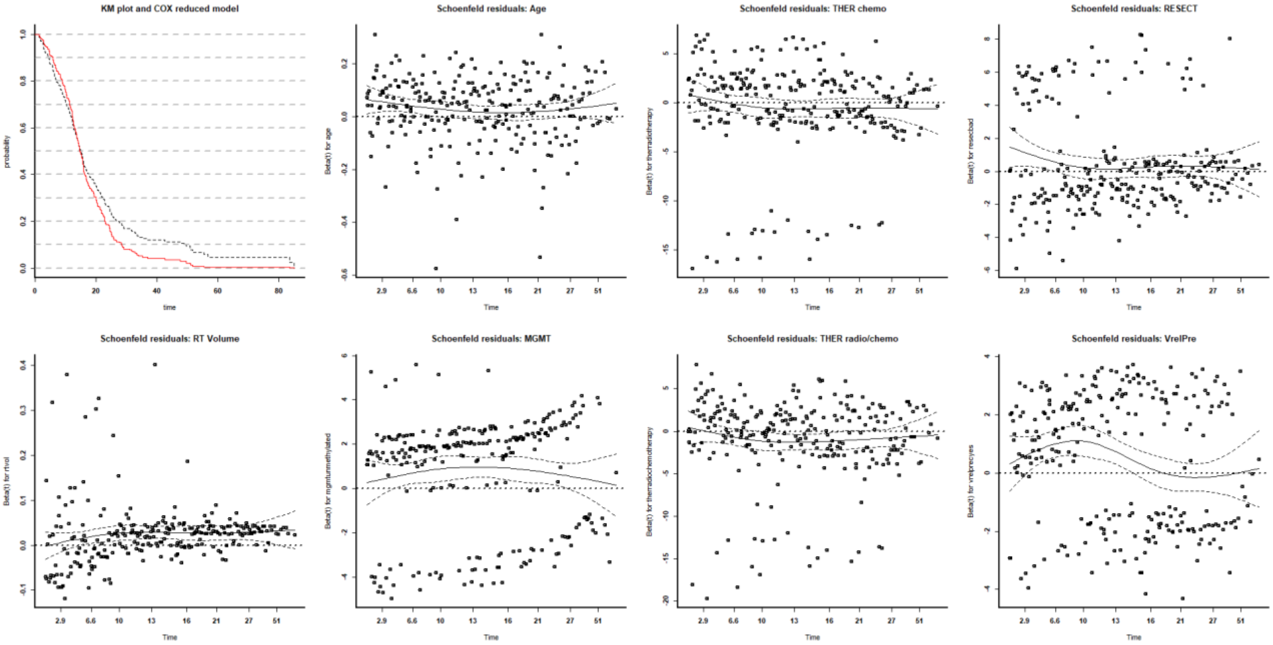

<u>k-fold cross-validation of Cox-model (internal):</u>

**index**	**orig**	**training**	**test**	**optimism**	**index corrected**	**n**
Dxy	0.4910	0.4915	0.4640	0.0275	**0.4635**	20
R^2	0.3922	0.3930	0.3702	0.0228	**0.3694**	20

1.3 Cox-Regression – reduced categorical model

For age class formation is made according to clinical aspects in three categories:

Age: ≤50 vs. >50 - ≤70 vs. >70

Classification thresholds for relative and absolute residual tumor volume were determined and the most appropriate thresholds were chosen, for details see 4. :

RTV: ≤10 cm^3 vs. >10 – ≤20cm^3 vs. >20 cm^3

The reduced categorical model is calculated including 6 predictors identified by univariable analyses:

	**coef**	**exp(coef)**	**se(coef)**	**z**	**Pr(>|z|)**
**rtv >10 – ≤20cm^3**	0.4997	1.6482	0.2907	1.719	0.08565
**rtv >20cm^3**	1.5782	4.8462	0.2547	6.196	5.78e-10 ***
**age >50 - ≤70**	0.4958	1.6418	0.2105	2.356	0.01848 *
**age >70**	0.8589	2.3605	0.2710	3.170	0.00153 **
**mgmt, unmethylated**	0.6715	1.9571	0.1510	4.446	8.76e-06 ***
**radiotherapy**	-0.4388	0.6448	0.2737	-1.603	0.10894
**radiochemotherapy**	-1.0332	0.3559	0.2919	-3.540	0.00040 ***
**resectability, bad**	0.3096	1.3629	0.1846	1.678	0.09341
**infiltration of vent. wall**	0.4462	1.5623	0.1409	3.166	0.00154 **

Estimated hazard ratios (95%-CI):

	**exp(coef)**	**exp(-coef)**	**lower .95**	**upper .95**
**rtv >10 – ≤20cm^3**	1.6482	0.6067	0.9323	2.9138
**rtv >20cm^3**	4.8462	0.2063	2.9417	7.9836
**age >50 - ≤70**	1.6418	0.6091	1.0869	2.4801
**age >70**	2.3605	0.4236	1.3879	4.0146
**mgmt, unmethylated**	1.9571	0.5110	1.4556	2.6314
**radiotherapy**	0.6448	1.5509	0.3771	1.1027
**radiochemotherapy**	0.3559	2.8100	0.2008	0.6306
**resectability, bad**	1.3629	0.7337	0.9492	1.9569
**infiltration of vent. wall**	1.5623	0.6401	1.1853	2.059

Concordance= 0.744 (se = 0.02)
R^2= 0.41

The class formation in the Cox regression model leads only to a rather slight change in C-index and in R^2. This model approach is therefore considered nearly equivalent and chosen because for the sake of simplicity for the further evaluations.

Test of proportional hazard assumption in Cox regression model:

	rho	chisq	p
**rtv >10 – ≤20cm^3**	-0.04126	0.47375	0.491
**rtv >20cm^3**	0.04490	0.56130	0.454
**age >50 - ≤70**	-0.04498	0.52146	0.470
**age >70**	-0.06654	1.08101	0.298
**mgmt, unmethylated**	-0.00406	0.00478	0.945

**radiotherapy**	-0.05473	0.89075	0.345
**radiochemotherapy**	-0.04991	0.69738	0.404
**resectability, bad**	-0.04048	0.47607	0.490
**infiltration of vent. wall**	-0.15665	6.46292	0.011

Schoenfeldt residual plots:

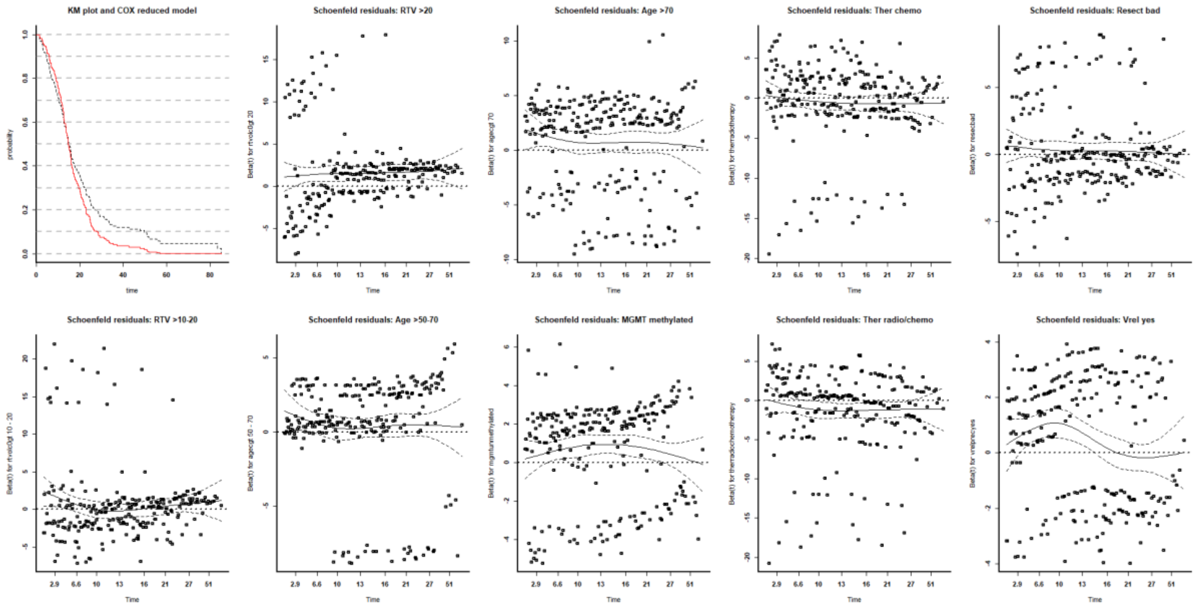

<u>k-fold cross-validation of Cox-model (internal):</u>

**index**	**orig**	**training**	**test**	**optimism**	**index corrected**	**n**
Dxy	0.4890	0.4890	0.4708	0.0182	**0.4635**	20
R^2	0.4099	0.3893	0.3993	-0.0100	**0.4199**	20

1.4 Log-logistic regression - reduced model

Loglogistic regression reduced model calculated including 6 predictors identified by univariable analyses:

		Model Likelihood Ratio Test		Discrimination Indexes	
Obs	303	LR chi2	156.41	R2	0.404
Events	254	d.f.	7	Dxy	0.496

	Coef	S.E.	Wald Z	Pr(>|Z|)
(Intercept)	4.1685	0.3978	10.48	<0.0001
**age**	-0.0183	0.0050	-3.69	0.0002
**rtv**	-0.0127	0.0032	-4.01	<0.0001
**mgmt, unmethylated**	-0.4316	0.0849	-5.08	<0.0001
**radiotherapy**	0.1295	0.1616	0.80	0.4229
**radiochemotherapy**	0.3884	0.1761	2.21	0.0274
**resectability, bad**	-0.3321	0.1095	-3.03	0.0024
**infiltration of vent. wall**	-0.3478	0.0823	-4.23	<0.0001
**Log(scale)**	-0.9653	0.0528	-18.29	<0.0001

The coefficients in the log-logistic model are to be interpreted differently than in the Cox regression. They detect acceleration or deceleration in survival times (acceleration factor, AF). The transformation with the exponential function leads to values <1 (delay - disadvantageous) or >1 (acceleration - advantageous). For example, the factor MGMT with exp (-0.4316) = 0.65 is associated with a survival time for unmethylated versus methylated shortened by a factor of 0.65. This survival time analysis approach provides a parametric (analytical) representation of the prediction / validation model in the following sections.

k-fold cross-validation of AFT-model (internal):

index	orig	training	test	optimism	index corrected	n
Dxy	0.4957	0.4954	0.4915	0.0040	**0.4917**	20
R^2	0.4038	0.4042	0.3878	0.0163	**0.3874**	20

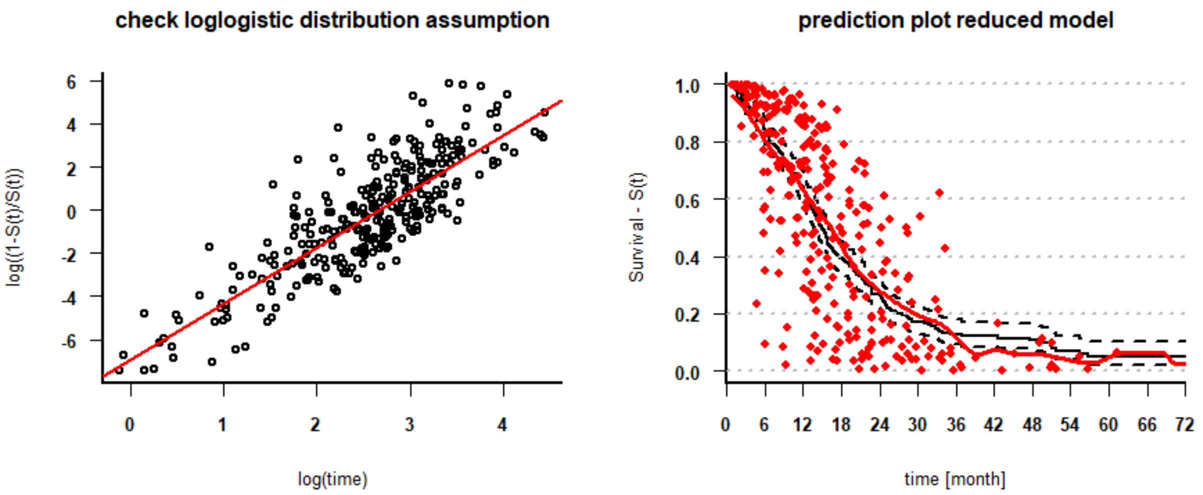

The hazard function in a log-logistic regression model is unimodal, which means the risk rises initially relatively steep and then steadily decreases. The acceptance of a log-logistic distribution is justified by a linearized transformation plot (left side). In the plot on the right side, individual survival probabilities are calculated based on the corresponding factor constellations with the model coefficients (red dots). The curve is then determined by a nonparametric regression (fit by a kernel density estimate - smoothing) and shows a relatively good approximation to the Kaplan-Meier estimate (on average).

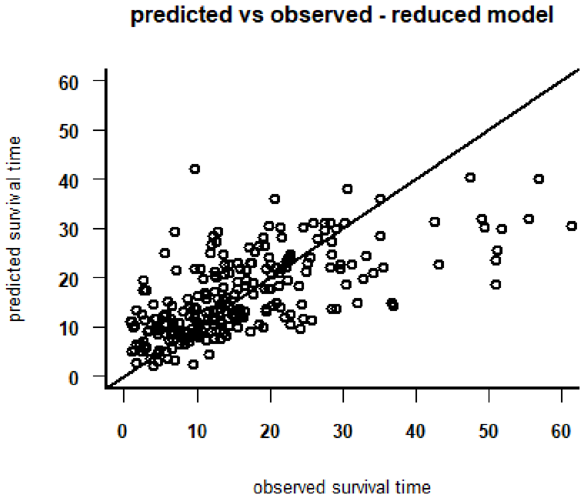

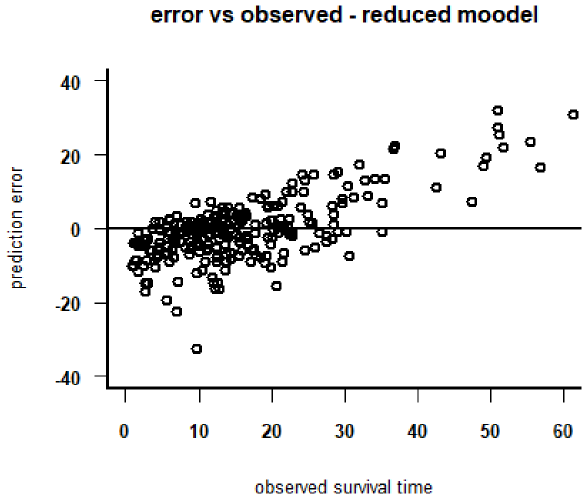

The residual plots shows the differences between observed and expected (model based) survival times. The mean deviation 0.31 months (median -1.2 months) is low, that is, the model applies to the observed data. However, individual deviations can be quite high and there is a trend towards underestimating longer survival!

<u>Residual Statistics:</u>

Min.	1st Qu.	Median	Mean	3rd Qu.	Max.	NA's
-32.52	-5.4520	-1.1880	0.3106	3.530	58.000	71

1.5 Log-logistic regression - reduced categorical model

<u>Loglogistic regression reduced categorical</u> model calculated including 6 predictors identified by univariable analyses:

		Model Likelihood Ratio Test		Discrimination Indexes	
Obs	303	LR chi2	166.09	R2	**0.423**
Events	254	d.f.	9	Dxy	**0.497**

	Coef	S.E.	Wald Z	Pr(>|Z|)
(Intercept)	3.2403	0.2046	15.84	<0.0001
**rtv >10 – ≤20cm^3**	-0.4717	0.1905	-2.48	0.0133
**rtv >20cm^3**	-0.7840	0.1498	-5.23	<0.0001
**age >50 - ≤70**	-0.3057	0.1169	-2.62	0.0089
**age >70**	-0.4798	0.1624	-2.95	0.0031
**mgmt, unmethylated**	-0.4131	0.0834	-4.95	<0.0001
**radiotherapy**	0.1512	0.1570	0.96	0.3356
**radiochemotherapy**	0.4905	0.1697	2.89	0.0039
**resectability, bad**	-0.2272	0.1066	-2.13	0.0330
**infiltration of vent. wall**	-0.3274	0.0810	-4.04	<0.0001
**Log(scale)**	-0.9845	0.0527	-18.69	<0.0001

k-fold cross-validation of AFT-model (internal):

Index	orig	training	test	optimism	index.corrected	n
Dxy	0.4971	0.4971	0.5106	-0.0135	**0.5106**	20
R^2	0.4226	0.4232	0.4286	-0.0054	**0.4280**	20

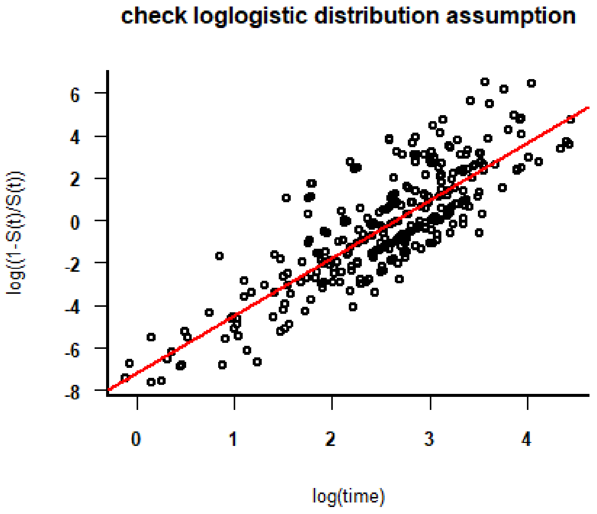

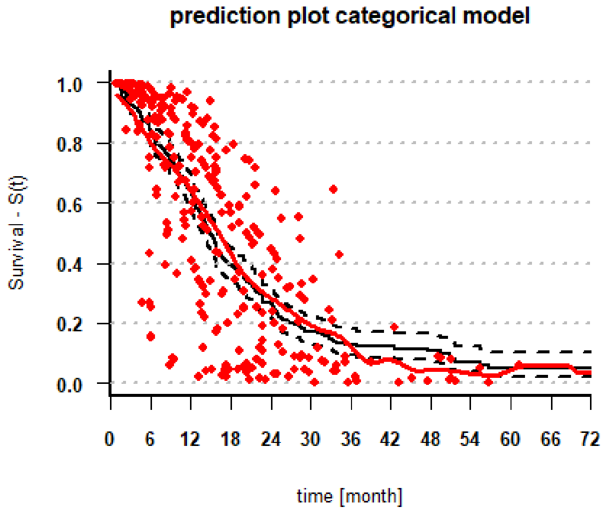

The hazard function in a log-logistic regression model is unimodal, which means the risk rises initially relatively steep and then steadily decreases. The acceptance of a log-logistic distribution is justified by a linearized transformation plot (left side). In the plot on the right side, individual survival probabilities are calculated based on the corresponding factor constellations with the model coefficients (red dots). The curve is then determined by a nonparametric regression (fit by a kernel density estimate - smoothing) and shows a relatively good approximation to the Kaplan-Meier estimate (on average).

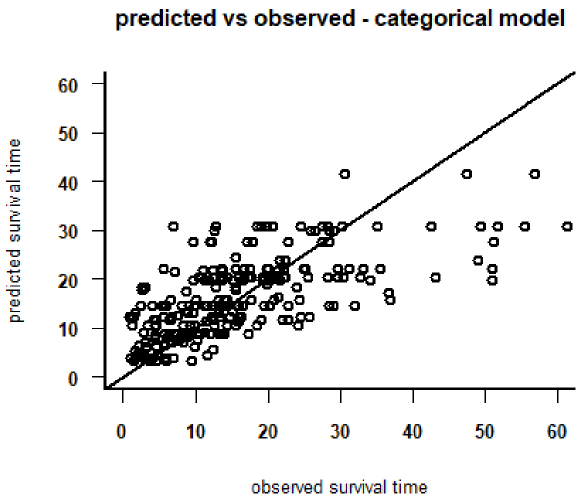

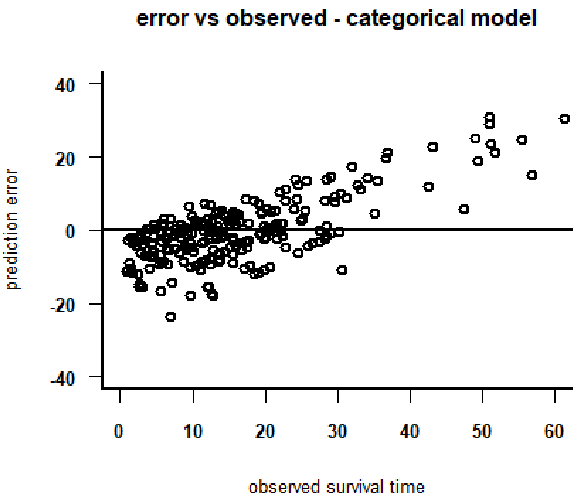

The residual plots shows the differences between observed and expected (model based) survival times. The mean deviation 0.30 months (median -0.95 months) is low, that is, the model applies to the observed data. However, individual deviations can be quite high and there is a trend towards underestimating longer survival!

Residual Statistics:

Min.	1st Qu.	Median	Mean	3rd Qu.	Max.	NA's
-23.76	-5.4310	-0.9446	0.2995	3.2320	61.5400	71

2. Scoring for survival

On the basis of the reduced categorical model (see section 1.5) the factors are weighted to a score by means from the following nomogram.

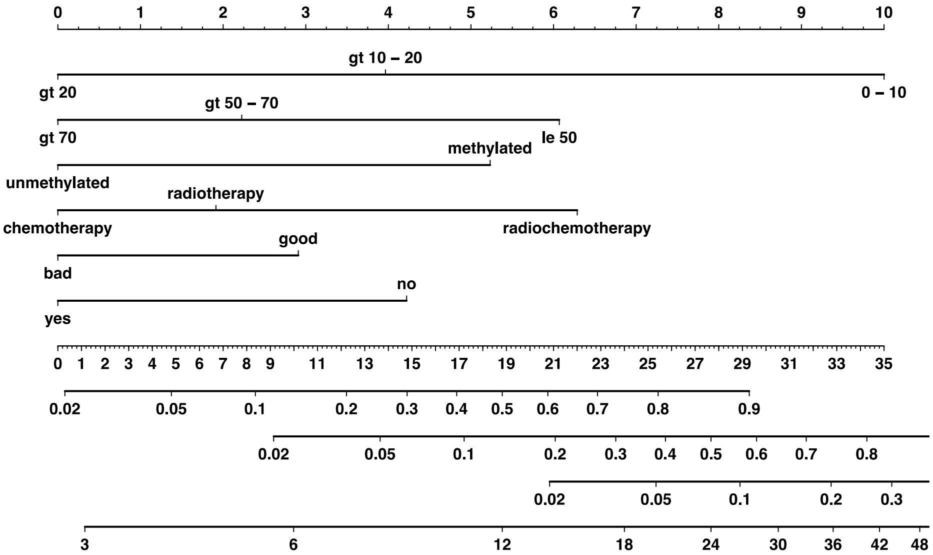

**Residual tumor volume (cm^3)**	≤10	>10 – ≤ 20cm^3	>20
	**10**	**4**	**0**
**Age (years)**	≤50	>50 - ≤ 70	>70
	**6**	**2**	**0**
**MGMT**	methylated		unmethylated
	**5**		**0**
**Therapy modality**	radiochemotherapy	radiotherapy	chemotherapy
	**6**	**2**	**0**
**Resectability**	good		bad
	**3**		**0**
**Infiltration of ventricular wall**	no		yes
	**4**		**0**

The 'worst' score (with the worst forecast) is thus 0, the best value 34.

Distribution of score values in modeling data set

Min.	1st Qu.	Median	Mean	3rd Qu.	Max.
2.00	17.00	21.00	20.76	25.00	34.00

2	4	5	6	7	8	9	10	11	12	13	14	15	17	18	19	20	21	22	23	24	25	26	27	29	30	34	Score
7	3	5	4	3	2	2	3	3	5	4	4	21	15	19	14	8	34	12	6	7	49	26	7	13	23	5	Patients

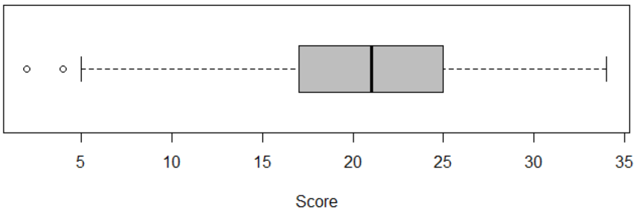

Loglogistic regression model calculated including score as only predictor:

		**Model Likelihood Ratio Test**		**Discrimination Indexes**	
Obs	304	LR chi2	166.95	**R^2**	**0.423**
Events	255	d.f.	1	**Dxy**	**0.496**

	**Coef**	**S.E.**	**Wald Z**	**Pr(>|Z|)**
(Intercept)	0.9820	0.1224	8.02	<0.0001
**score**	0.0811	0.0056	14.53	<0.0001
**Log(scale)**	-0.9856	0.0525	-18.78	<0.0001

**Index**	**orig**	**training**	**test**	**optimism**	**index corrected**	**n**
Dxy	0.4957	0.4953	0.5011	0.0057	**0.5014**	20
R^2	0.4231	0.4229	0.4288	0.0059	**0.4291**	20

The score obviously describes the influence of the investigated factors very well. The corresponding log-logistic regression model shows comparable results to the model from section 1.5 and is therefore additionally taken into account in the validation of the model.

Prediction from the coefficients of the model

For the selected factors (factor combinations) predictions for the survival time with the model coefficients are calculated according to the model from section 1.4 and for the score from section 2. The representations can also be transferred to other factors according to clinical considerations.

Prediction of the log-logistic score-model

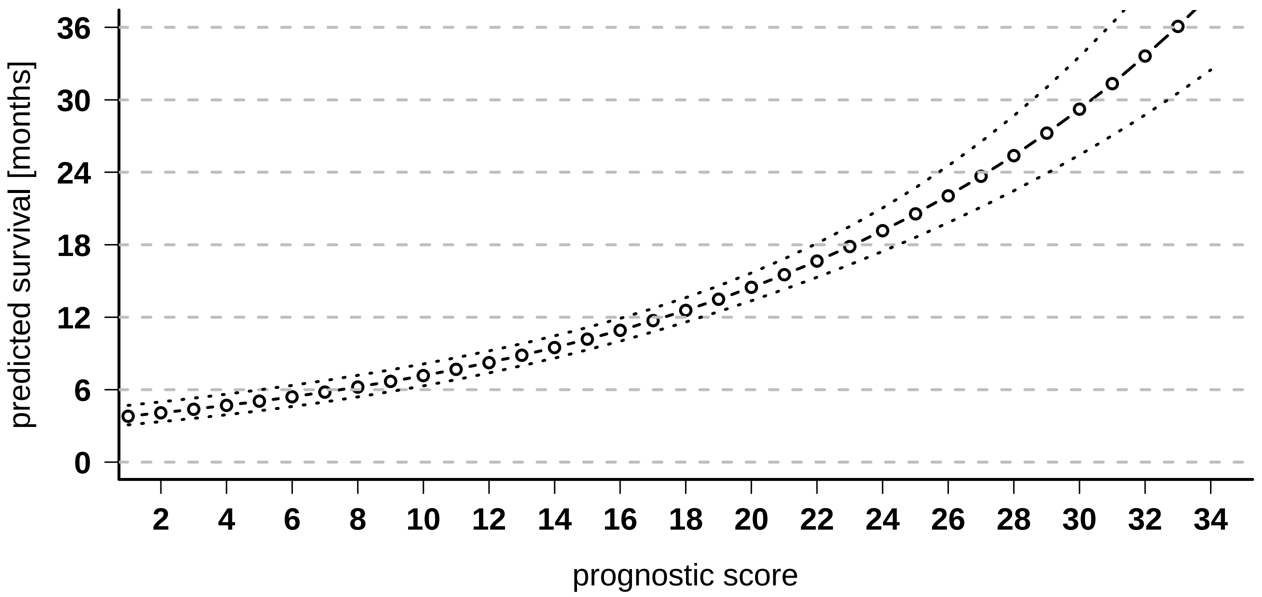

3. Model validation on external data

After the previous analyzes, the validation takes place on the basis of the log-logistic model with categorical influencing variables and additionally for the derived score.
For each validation step, a new (external) model of the selected factors is first calculated. This is followed by a prediction with the coefficients from the reference model (Section 1) and an evaluation of the residuals. The estimate is not bad in the 'mean'. However, there is an obvious scattering of the residuals and it shows a systematic error by underestimating the higher survival times!

3.1 Validation of external patient cohort

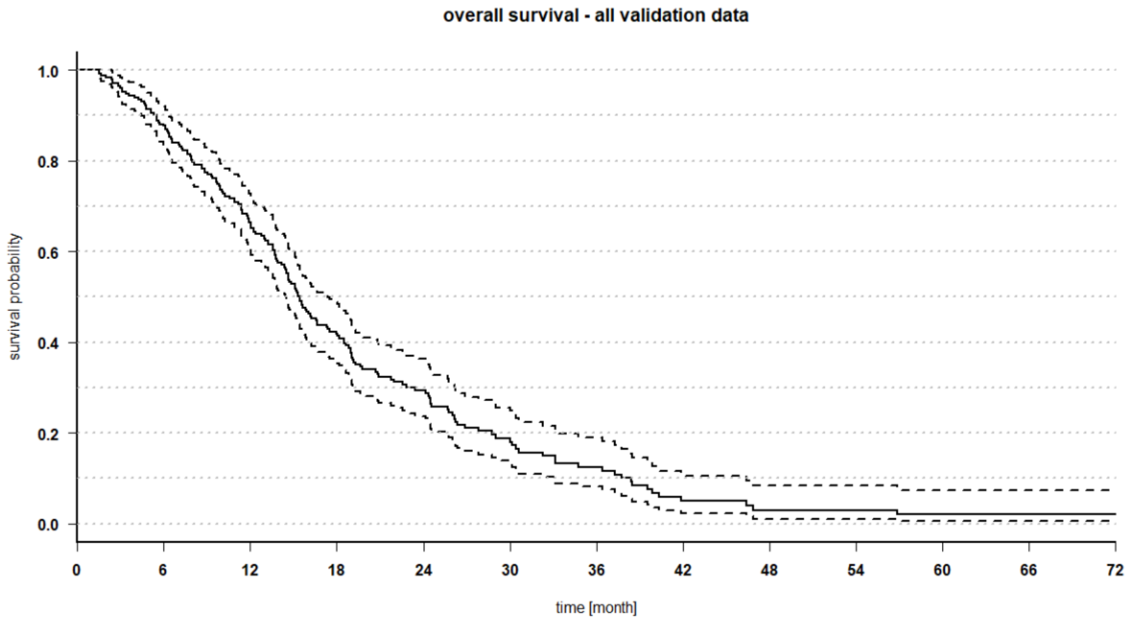

Log logistic regression model (reduced, categorical) from validation data:

	Model Likelihood Ratio Test			Discrimination Indexes	
Obs	253	LR chi2	79.70	R^2	**0.271**
Events	191	d.f.	9	Dxy	**0.372**

	Coef	S.E.	Wald Z	Pr(>|Z|)
(Intercept)	3.2181	0.2010	16.01	<0.0001
**rtv >10 – ≤20cm³**	-0.1797	0.3107	-0.58	0.5630
**rtv >20cm³**	-0.4859	0.2033	-2.39	0.0169
**age >50 - ≤70**	-0.1043	0.1218	-0.86	0.3918
**age >70**	-0.4825	0.1550	-3.11	0.0018
**mgmt, unmethylated**	-0.3826	0.0959	-3.99	<0.0001
**radiotherapy**	-0.1169	0.1815	-0.64	0.5196
**radiochemotherapy**	0.2617	0.1642	1.59	0.1109
**resectability, bad**	-0.1003	0.1985	-0.51	0.6132
**infiltration of vent. wall**	-0.3407	0.0943	-3.61	0.0003
**Log(scale)**	-0.9825	0.0593	-16.58	<0.0001

**Index**	**orig**	**training**	**test**	**optimism**	**Index corrected**	**n**
Dxy	0.3720	0.3860	0.3558	0.0302	**0.3418**	20
R^2	0.2710	0.2924	0.2464	0.0460	**0.2250**	20

The estimation (prediction) of the survival times in the validation cohorts with the coefficients of selected factors from modelling dataset shows the following distribution of residuals (error).

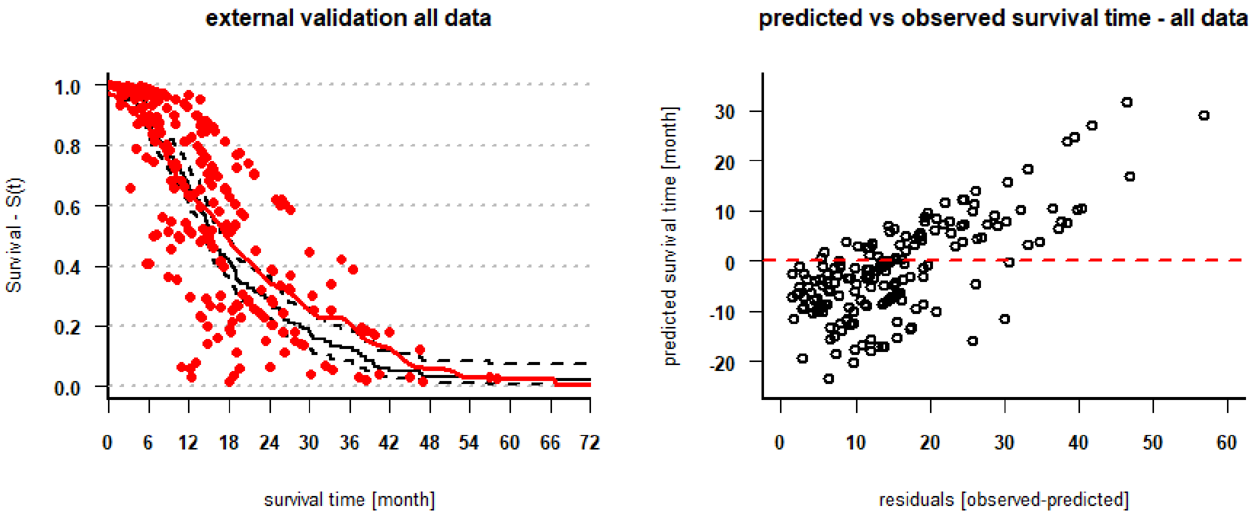

The external data used here for validation show a different result for the selected factors, i.e. their composition has to be evaluated differently...!

<u>Residual Statistics:</u>

**Min.**	**1st Qu.**	**Median**	**Mean**	**3rd Qu.**	**Max.**
-23.550	-7.878	-2.634	-1.808	4.027	31.73

Log logistic regression model (score model) from validation data:

		Model Likelihood Ratio Test		Discrimination Indexes	
Obs	253	LR chi2	68.95	$R2$	**0.239**
Events	191	d.f.	1	Dxy	**0.357**

	**Coef**	**S.E.**	**Wald Z**	**Pr(>|Z|)**
(Intercept)	1.1102	0.1850	6.00	<0.0001
**scorec**	0.0715	0.0080	8.90	<0.0001
**Log(scale)**	-0.9608	0.0592	-16.23	<0.0001

**Index**	**orig**	**training**	**test**	**optimism**	**index corrected**	**n**
Dxy	0.3568	0.3551	0.3568	-0.0017	**0.3585**	20
R^2	0.2392	0.2386	0.2392	-0.0006	**0.2399**	20

The estimation (prediction) of the survival times in the validation cohorts with the coefficient for the score factor from modelling dataset shows the following distribution of residuals (error).

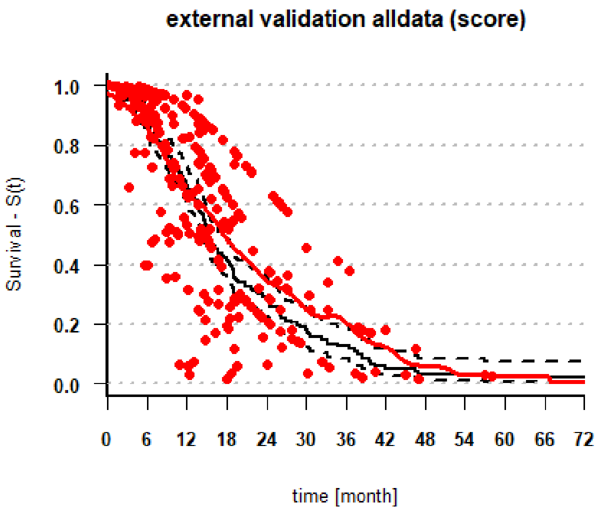

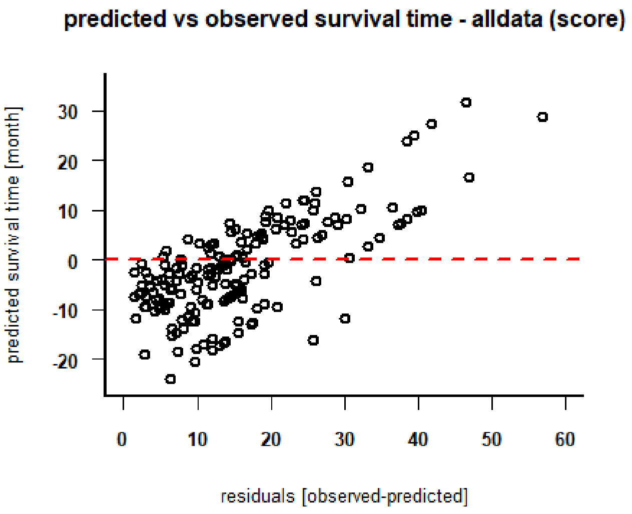

The score summarized the informations from the selected factors very well. The resulting residuals are very simular to the above distribution. Nevertheless predicted survival is biased. Maybe the validation cohorts differs in baseline characteristics and/or measurement criteria.

Residual Statistics:

**Min.**	**1st Qu.**	**Median**	**Mean**	**3rd Qu.**	**Max.**
-23.980	-8.035	-2.782	-1.839	4.252	31.780

4. Appendix - Comparison of different EOR models

4.1 Current model (categorical model – 3 resection categories, absolute)

287 cases (239 events) are available in total; compared to the previous model analyses, there are also cases that do not show the remaining volume as a percentage, because of missing preoperative tumor volume (n=16). The current model used here for comparison different models therefore differs slightly from final model but allows a direct comparison between all absolute and relative EOR models!

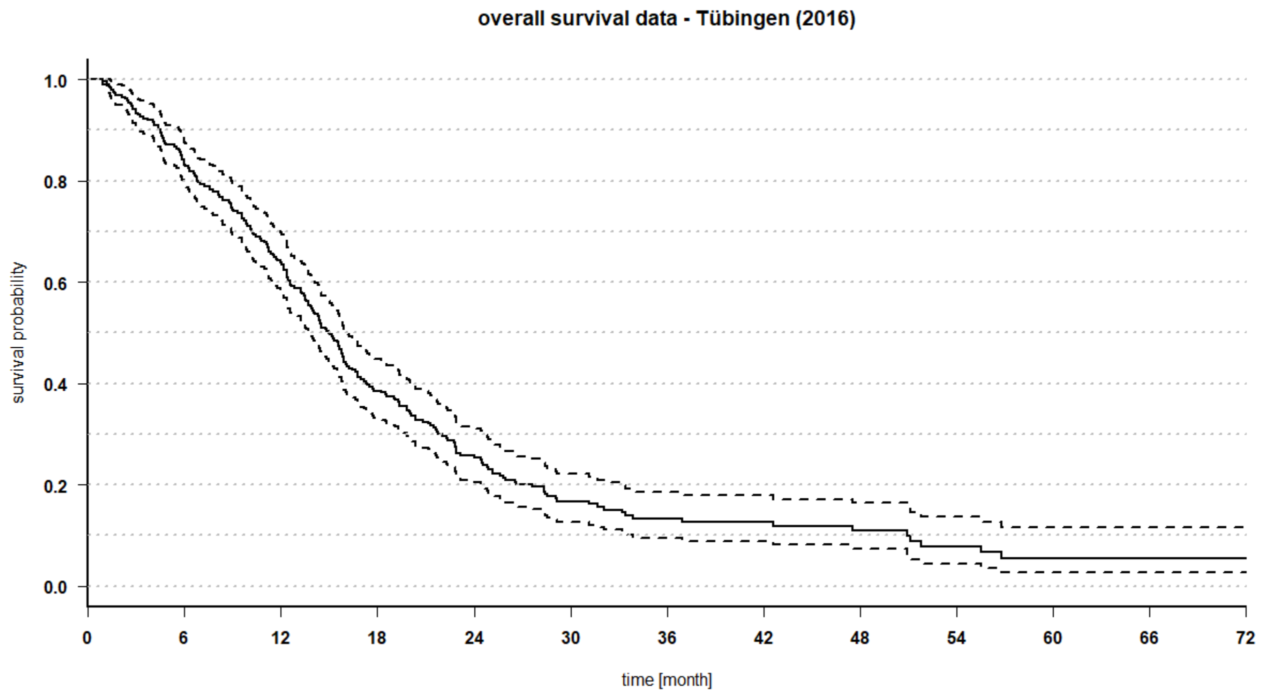

The null model describes the variability (uncertainty) in the survival times without consideration of influencing variables and serves as a reference for the evaluation of the following regression models.
---> COX regression modeling: null model

Null model log likelihood= -1151.87 n= 287

Cox regression model with EOR in 3 categories; reference category is 0-10cm^3:

	**coef**	**exp(coef)**	**se(coef)**	**z**	**Pr(>|z|)**
**age >50 - ≤70**	0.4743	1.6070	0.2118	2.240	0.025087 *
**age >70**	0.8191	2.2685	0.2802	2.923	0.003467 **
**mgmt, unmethylated**	0.7354	2.0863	0.1563	4.705	2.53e-06 ***
**radiotherapy**	-0.5355	0.5854	0.2729	-1.962	0.049733 *
**radiochemotherapy**	-1.1106	0.3293	0.2983	-3.724	0.000196 ***
**infiltration of vent. wall**	0.5264	1.6929	0.1411	3.731	0.000191 ***
**rtv >10 – ≤20cm^3**	0.5797	1.7856	0.2806	2.0661	0.038851 *
**rtv >20cm^3**	1.7458	5.7303	0.2483	7.031	2.05e-12 ***

Estimated hazard ratios (95% CI):

	exp(coef)	exp(-coef)	lower .95	upper .95
**age >50 - ≤70**	1.6070	0.6223	1.0611	2.4336
**age >70**	2.2685	0.4408	1.3098	3.9289
**mgmt, unmethylated**	2.0863	0.4793	1.5358	2.8341
**radiotherapy**	0.5854	1.7083	0.3429	0.9994
**radiochemotherapy**	0.3293	3.0363	0.1836	0.5909
**infiltration of vent. wall**	1.6929	0.5907	1.2839	2.2322
**rtv >10 – ≤20cm^3**	1.7856	0.5600	1.0301	3.0950
**rtv >20cm^3**	5.7303	0.1745	3.5223	9.3225

Concordance= 0.744 (se = 0.021)
R^2= 0.411 (max possible= 1)

4.2 Categorical model – 2 resection categories, absolute

Cox regression model with EOR in 2 categories; reference category is 0cm³:

	coef	exp(coef)	se(coef)	z	Pr(>|z|)
**age >50 - ≤70**	0.5392	1.7147	0.2113	2.551	0.010726 *
**age >70**	0.9370	2.5524	0.2798	3.349	0.000810 ***
**mgmt, unmethylated**	0.7644	2.1476	0.1567	4.877	1.08e-06 ***
**radiotherapy**	-0.3708	0.6902	0.2706	-1.370	0.170594
**radiochemotherapy**	-1.0114	0.3637	0.2973	-3.402	0.000669 ***
**infiltration of vent. wall**	0.5377	1.7120	0.1408	3.819	0.000134 ***
**rtv >0cm³**	0.1936	1.2136	0.1598	1.211	0.225829

Estimated hazard ratios (95% CI):

	exp(coef)	exp(-coef)	lower .95	upper .95
**age >50 - ≤70**	1.7147	0.5832	1.1332	2.5946
**age >70**	2.5524	0.3918	1.4751	4.4165
**mgmt, unmethylated**	2.1476	0.4656	1.5796	2.9199
**radiotherapy**	0.6902	1.4489	0.4061	1.1730
**radiochemotherapy**	0.3637	2.7496	0.2031	0.6513
**infiltration of vent. wall**	1.7120	0.5841	1.2991	2.2561
**rtv >0cm³**	1.2136	0.8240	0.8872	1.6600

Concordance= 0.726 (se = 0.021)
R^2= 0.329 (max. possible= 1)

4.3 Categorical model – 2 resection categories, absolute

Cox regression model with EOR in 2 categories; reference category is <=1cm³:

	coef	exp(coef)	se(coef)	z	Pr(>|z|)
**age >50 - ≤70**	0.4278	1.5339	0.2116	2.022	0.043203 *
**age >70**	0.8997	2.4589	0.2793	3.221	0.001276 **
**mgmt, unmethylated**	0.7321	2.0795	0.1566	4.675	2.94e-06 ***
**radiotherapy**	-0.2913	0.7473	0.2705	-1.077	0.281570
**radiochemotherapy**	-0.9177	0.3994	0.2977	-3.082	0.002056 **
**infiltration of vent. wall**	0.5553	1.7424	0.1394	3.983	6.81e-05 ***
**rtv >1 cm³**	0.4976	1.6448	0.1346	3.697	0.000218***

Estimated hazard ratios (95% CI):

	exp(coef)	exp(-coef)	lower .95	upper .95
**age >50 - ≤70**	1.5339	0.6519	1.0132	2.322
**age >70**	2.4589	0.4067	1.4224	4.251
**mgmt, unmethylated**	2.0795	0.4809	1.5299	2.827
**radiotherapy**	0.7473	1.3382	0.4398	1.270
**radiochemotherapy**	0.3994	2.5035	0.2229	0.716
**infiltration of vent. wall**	1.7424	0.5739	1.3258	2.290
**rtv >1 cm³**	1.6448	0.6080	1.2634	2.141

Concordance= 0.736 (se = 0.021)
R^2= 0.356 (max possible= 1)

4.4 Categorical model – 3 resection categories, absolute

Cox regression model with EOR in 3 categories; reference category is <=1cm³:

	coef	exp(coef)	se(coef)	z	Pr(>|z|)
**age >50 - ≤70**	0.4378	1.5493	0.2124	2.061	0.03931 *
**age >70**	0.8657	2.3767	0.2807	3.084	0.00204 **
**mgmt, unmethylated**	0.6967	2.0071	0.1571	4.433	9.27e-06 ***
**radiotherapy**	-0.4116	0.6626	0.2739	-1.503	0.13295
**radiochemotherapy**	-0.9124	0.4015	0.2992	-3.050	0.00229 **
**infiltration of vent. wall**	0.5316	1.7016	0.1402	3.790	0.00015 ***
**rtv >1 – ≤10cm³**	0.2904	1.3370	0.1499	1.937	0.05277 .
**rtv >10 cm³**	1.2152	3.3710	0.2062	5.894	3.77e-09 ***

Estimated hazard ratios (95% CI):

	exp(coef)	exp(-coef)	lower .95	upper .95
**age >50 - ≤70**	1.5493	0.6454	1.0217	2.3495
**age >70**	2.3767	0.4207	1.3711	4.1201
**mgmt unmethylated**	2.0071	0.4982	1.4751	2.7311
**radiotherapy**	0.6626	1.5093	0.3873	1.1335
**radiochemotherapy**	0.4015	2.4904	0.2234	0.7217
**infiltration of vent. wall**	1.7016	0.5877	1.2927	2.2399
**rtv >1 – ≤10cm³**	1.3370	0.7480	0.9965	1.7937
**rtv >10 cm³**	3.3710	0.2967	2.2504	5.0495

Concordance= 0.745 (se = 0.021)
R^2= 0.393 (max possible= 1)

4.5 Categorical model – 3 resection categories, absolute

Cox regression model with EOR in 3 categories; reference category is 0cm^3:

	coef	exp(coef)	se(coef)	z	Pr(>|z|)
**age >50 - ≤70**	0.4088	1.5051	0.2131	1.918	0.05507 .
**age >70**	0.8813	2.4141	0.2799	3.149	0.00164 **
**mgmt, unmethylated**	0.7273	2.0695	0.1564	4.650	3.32e-06 ***
**radiotherapy**	-0.2752	0.7594	0.2709	-1.016	0.30977
**radiochemotherapy**	-0.9192	0.3988	0.2973	-3.092	0.00199 **
**infiltration of vent. wall**	0.5733	1.7741	0.1419	4.040	5.34e-05 ***
**rtv >0 - ≤1cm^3**	-0.1319	0.8765	0.1900	-0.694	0.48763
**rtv >1cm^3**	0.4241	1.5282	0.1696	2.500	0.01242 *

Estimated hazard ratios (95% CI):

	exp(coef)	exp(-coef)	lower .95	upper .95
**age >50 - ≤70**	1.5051	0.6644	0.9912	2.2854
**age >70**	2.4141	0.4142	1.3949	4.1779
**mgmt, unmethylated**	2.0695	0.4832	1.5231	2.8118
**radiotherapy**	0.7594	1.3168	0.4465	1.2915
**radiochemotherapy**	0.3988	2.5073	0.2227	0.7143
**infiltration of vent. wall**	1.7741	0.5637	1.3434	2.3429
**rtv >0 - ≤1cm^3**	0.8765	1.1410	0.6040	1.2719
**rtv >1cm^3**	1.5282	0.6543	1.0960	2.1310

Concordance= 0.735 (se = 0.021)
R^2= 0.358 (max possible= 1)

4.6 Categorical model – 5 resection categories, absolute

Cox regression model with EOR in 5 categories; reference category is 0cm^3:

	coef	exp(coef)	se(coef)	z	Pr(>|z|)
**age >50 - ≤70**	0.4101	1.5069	0.2145	1.912	0.055924 .
**age >70**	0.8380	2.3118	0.2819	2.972	0.002957 **
**mgmt, unmethylated**	0.6894	1.9925	0.1572	4.385	1.16e-05 ***
**radiotherapy**	-0.4127	0.6619	0.2755	-1.498	0.134239
**radiochemotherapy**	-0.9143	0.4008	0.2984	-3.064	0.002185**
**infiltration of vent. wall**	0.5485	1.7307	0.1425	3.849	0.000119***
**rtv >0 - ≤0.2cm^3**	-0.2174	0.8046	0.2498	-0.870	0.384188
**rtv >0.2 - ≤1 cm^3**	-0.0252	0.9751	0.2116	-0.119	0.905168
**rtv >1 - ≤10cm^3**	0.2372	1.2678	0.1808	1.312	0.189410
**rtv >10cm^3**	1.1651	3.2061	0.2346	4.967	6.80e-07 ***

Estimated hazard ratios (95% CI):

	exp(coef)	exp(-coef)	lower .95	upper .95
**age >50 - ≤70**	1.5069	0.6636	0.9897	2.2945
**age >70**	2.3118	0.4326	1.3303	4.0174
**mgmt, unmethylated**	1.9925	0.5019	1.4642	2.7115
**radiotherapy**	0.6619	1.5108	0.3857	1.1359
**radiochemotherapy**	0.4008	2.4949	0.2233	0.7194
**infiltration of vent. wall**	1.7307	0.5778	1.3089	2.2885
**rtv >0 - ≤0.2cm^3**	0.8046	1.2428	0.4931	1.3129
**rtv >0.2 - ≤1 cm^3**	0.9751	1.0255	0.6441	1.4762
**rtv >1 - ≤10cm^3**	1.2678	0.7888	0.8895	1.8068
**rtv >10cm^3**	3.2061	0.3119	2.0245	5.0773

Concordance= 0.746 (se = 0.021)
R^2= 0.395 (max possible= 1)

4.7 Categorical model – 2 resection categories, relative in percent

Cox regression model with EOR (%) in 2 categories; reference category is <=98%:

	coef	exp(coef)	se(coef)	z	Pr(>|z|)
**age >50 - ≤70**	0.5228	1.6868	0.2106	2.483	0.01304 *
**age >70**	0.8589	2.3605	0.2796	3.072	0.00213 **
**mgmt, unmethylated**	0.7181	2.0506	0.1579	4.547	5.45e-06 ***
**radiotherapy**	-0.3535	0.7022	0.2745	-1.288	0.19788
**radiochemotherapy**	-0.9186	0.3991	0.3025	-3.037	0.00239 **
**infiltration of vent. wall**	0.6184	1.8560	0.1395	4.434	9.27e-06 ***
**rtv >98%**	0.7064	2.0267	0.1747	4.043	5.28e-05 ***

Estimated hazard ratios (95% CI):

	exp(coef)	exp(-coef)	lower .95	upper .95
**age >50 - ≤70**	1.6868	0.5928	1.1163	2.5488
**age >70**	2.3605	0.4236	1.3646	4.0832
**mgmt, unmethylated**	2.0506	0.4877	1.5046	2.7946
**radiotherapy**	0.7022	1.4241	0.4100	1.2027
**radiochemotherapy**	0.3991	2.5059	0.2206	0.7219
**infiltration of vent. wall**	1.8560	0.5388	1.4120	2.4395
**rtv >98%**	2.0267	0.4934	1.4390	2.8544

Concordance= 0.742 (se = 0.021)
R^2= 0.359 (max possible= 1)

4.8 Categorical model – 3 resection categories, relative in percent

Cox regression model with EOR (%) in 3 categories; reference category is 0 %:

	coef	exp(coef)	se(coef)	z	Pr(>|z|)
**age >50 - ≤70**	0.52746	1.69462	0.21118	2.498	0.012503 *
**age >70**	0.86722	2.38028	0.28124	3.084	0.002046 **
**mgmt, unmethylated**	0.72074	2.05596	0.15831	4.553	5.29e-06 ***
**radiotherapy**	-0.35763	0.69933	0.27504	-1.300	0.193496
**radiochemotherapy**	-0.91511	0.40047	0.30279	-3.022	0.002509 **
**infiltration of vent. wall**	0.61066	1.84165	0.14188	4.304	1.68e-05 ***
**rtv >0% - ≤95%**	0.04997	1.05124	0.16614	0.301	0.763595
**rtv >95%**	0.74124	2.09854	0.21007	3.529	0.000418***

Estimated hazard ratios (95% CI):

	exp(coef)	exp(-coef)	lower .95	upper .95
**age >50 - ≤70**	1.6946	0.5901	1.1202	2.5635
**age >70**	2.3803	0.4201	1.3716	4.1307
**mgmt, unmethylated**	2.0560	0.4864	1.5075	2.8039
**radiotherapy**	0.6993	1.4299	0.4079	1.1989
**radiochemotherapy**	0.4005	2.4970	0.2212	0.7249
**infiltration of vent. wall**	1.8417	0.5430	1.3946	2.4321
**rtv >0% - ≤95%**	1.0512	0.9513	0.7591	1.4559
**rtv >95%**	2.0985	0.4765	1.3903	3.1676

Concordance= 0.743 (se = 0.021)
R^2= 0.359 (max possible= 1)

4.9 Categorical model – 2 resection categories, relative in percent

In this model the EOR is considered relatively (in %) in 2 categories!
Reference category is <=80%

Cox regression model with EOR (%) in 2 categories; reference category is <=80 %:

	coef	exp(coef)	se(coef)	z	Pr(>|z|)
**age >50 - ≤70**	0.5228	1.6868	0.2106	2.483	0.01304 *
**age >70**	0.8589	2.3605	0.2796	3.072	0.00213 **
**mgmt, unmethylated**	0.7181	2.0506	0.1579	4.547	5.45e-06 ***
**radiotherapy**	-0.3535	0.7022	0.2745	-1.288	0.19788
**radiochemotherapy**	-0.9186	0.3991	0.3025	-3.037	0.00239 **
**infiltration of vent. wall**	0.6184	1.8560	0.1395	4.434	9.27e-06 ***
**rtv <80 %**	0.7064	2.0267	0.1747	4.043	5.28e-05 ***

Estimated hazard ratios (95% CI):

	exp(coef)	exp(-coef)	lower .95	upper .95
**age >50 - ≤70**	1.6868	0.5928	1.1163	2.5488
**age >70**	2.3605	0.4236	1.3646	4.0832
**mgmt, unmethylated**	2.0506	0.4877	1.5046	2.7946
**radiotherapy**	0.7022	1.4241	0.4100	1.2027
**radiochemotherapy**	0.3991	2.5059	0.2206	0.7219
**infiltration of vent. wall**	1.8560	0.5388	1.4120	2.4395
**rtv <80 %**	2.0267	0.4934	1.4390	2.8544

Concordance= 0.742 (se = 0.021)
R^2= 0.359 (max possible= 1)

Summary of resection thresholds

Thresholds	r^2	Concordance index
0% vs >0%;	R2=0.329,	C=0.726 (se=0.02)
<=98% vs >98%	R2=0.359,	C=0.742 (se=0.02)
0% vs >0% - 95% vs < 95%	R2=0.359,	C=0.743 (se=0.02)
<=80% vs >80%	R2=0.359,	C=0.742 (se=0.02)
0 vs >0	R2=0.329,	C=0.726 (se=0.02)
<=1 vs > 1	R2=0.356,	C=0.736 (se=0.02)
0 vs 0 - <=1 vs > 1	R2=0.358,	C=0.735 (se=0.02)
<=1 vs >1 - <=10 vs > 10	R2=0.393,	C=0.745 (se=0.02)
0 vs 0 - <=0.2 vs 0.2 - <=1 vs 1-<=10 vs> 10	R2=0.395,	C=0.746 (se=0.02)
**0- <=10 vs <10 - <=20 vs > 20**	**R2=0.411,**	**C=0.744 (se=0.02)**

Supplementary File 2

Demographic and clinical data	N	% of all
**Gender**		
female	141	47 %
male	162	53 %
**Age**		
Median	62	
Quantile_10	48	
Quantile_90	77	
Mean	62	
SD	12	
≤50	44	14 %
50-70	175	58 %
>70	84	28 %
**Resection**		
Gross Total	162	54 %
Subtotal	86	28 %
Biopsy	55	18 %
**Residual tumor volume (in cm^3)**		
Median	0.7	
Quantile_10	0	
Quantile_90	17	
Mean	6	
SD	14	
0	90	30 %
>0-≤1	75	25 %
>1-≤10	95	31 %
>10	43	14 %
**Extent of resection (% residual tumor)**		
Median	3	
Quantile_10	0	
Quantile_90	100	
Mean	24	
SD	38	
0	90	30 %
>0 - ≤5	77	25 %
>5 - ≤20	48	16 %
>20	72	24 %
missing	16	5 %
**Resectability**		

Supplementary File 2

Demographic and clinical data	N	% of all
good	252	83 %
bad	51	17 %
**Tumor reaching/infiltrating ventricular wall**		
No	123	41 %
Yes	180	59 %
**Side**		
right	143	47 %
left	146	48 %
bilateral	14	5 %
**Tumor localization**		
frontal	50	16%
precentral	14	5 %
postcentral	5	2 %
central (precentral & postcentral)	16	5 %
fronto-temporal	9	3 %
parietal	19	6 %
parieto-temporal	13	4 %
parieto-occipital	9	3 %
temporo-fronto-insular	16	5 %
temporo-occipital	12	4 %
occipital	5	2 %
diencephalic	11	4 %
brain stem	1	<1 %
> 2 lobes	19	6 %
multicentric	28	9 %
temporal	61	20 %
insular	4	1 %
basal ganglia	6	2 %
thalamic	5	2 %
**Eloquence**		
not eloquent	207	68 %
central (motoric/sensoric)	39	13 %
Broca’s speech area	13	4 %
Wernicke's speech area	15	5 %
Infer.parietal lobule “Geschwind’s” region	12	4 %
primary visual cortex	16	5 %
missing	1	<1 %
**Karnofsky performance scale**		

Supplementary File 2

**Demographic and clinical data**	**N**	**% of all**
	**N**	**% of all**
Median	80	
Quantile_10	60	
Quantile_90	90	
Mean	74	
SD	15	
100%	25	8 %
90%	99	33 %
80%	99	33 %
70%	39	13 %
<70	16	13 %
**Therapy modality**		
Radiotherapy (RT)	112	37 %
Gray, median (IQ 10-90)	39 (34-60) Gy	
Latency to RT, median (IQ 10-90)	31 (17-48) days	
Chemotherapy with TMZ	20	7 %
Radiochemotherapy with TMZ	171	56 %
Gray, median (IQ 10-90)	60 (59,4-60) days	
Latency to RT, median (IQ 10-90)	28 (18-39) Gy	
**MGMT**		
methylated	107	35 %
unmethylated	196	65 %
**Preoperative steroids**		
no	203	67 %
yes	95	31 %
missing	5	2 %
**Postoperative new deficits**		
no	237	78 %
yes	49	16 %
missing	17	6 %
**Recurrent surgery**		
yes	57	19 %
no	148	49 %
missing	98	32 %

Supplementary File 3

Overall survival Kaplan Meier estimates

time (months)	patient at risk	patient events	survival	standard error	lower 95% CI	upper 95% CI
0	304	0	1.0000	0.0000	1.0000	1.000
6	255	50	0.8345	0.0214	0.7936	0.877
12	189	57	0.6419	0.0278	0.5897	0.699
18	111	72	0.3928	0.0286	0.3406	0.453
24	66	34	0.2642	0.0265	0.2171	0.322
30	36	22	0.1725	0.0235	0.1321	0.225
36	20	9	0.1261	0.0217	0.0900	0.177
42	17	1	0.1195	0.0215	0.0839	0.170
48	13	2	0.1044	0.0213	0.0700	0.156
54	7	4	0.0677	0.0203	0.0376	0.122
60	5	2	0.0483	0.0185	0.0228	0.103

Supplementary File 4

Nomogram predicted survival versus actual survival

PATIENT 1

Predictors	Category	Points
**Predictors**	**Category**	**Points**
RTV	2.69 cm^3	10
Age	70	2
MGMT	unmethylated	0
Therapy	Chemotherapy	0
Resectability	good	3
Infiltration		
Ventricle wall	yes	0
Total points		15
**Predicted survival**		11 months
**Actual Survival**		12 months

PATIENT 2

Predictors	Category	Points
RTV	2.68 cm^3	10
Age	52	2
MGMT	methylated	5
Therapy	Radiochemo	6
Resectability	good	3
Infiltration		
Ventricle wall	no	4
Total points		30
**Predicted survival**		28 months
**Actual Survival**		24 months

PATIENT 3

Predictors	Category	Points
RTV	18.09 cm^3	4
Age	80	0
MGMT	unmethylated	0
Therapy	Chemotherapy	0
Resectability	good	3
Infiltration		
Ventricle wall	no	4
Total points		11
**Predicted survival**		8 months
**Actual Survival**		7 months

PATIENT 4

Predictors	Category	Points
RTV	11.69 cm^3	4
Age	51	2
MGMT	methylated	5
Therapy	Radiochemo	6
Resectability	good	3
Infiltration		
Ventricle wall	no	4
Total points		24
**Predicted survival**		19 months
**Actual Survival**		34 months
